# Crosslinker identity and dynamics regulate F-actin network structure and mechanics

**DOI:** 10.3389/fcell.2026.1900481

**Published:** 2026-08-28

**Authors:** Lauren Godfrey, Bekele J. Gurmessa

**Affiliations:** Department of Physics and Astronomy, Bucknell University, Lewisburg, PA, United States

**Keywords:** actin crosslinkers, biotin–NeutrAvidin, F-actin networks, nonlinear mechanics, microrheology, stress relaxation, viscoelasticity, α-actinin

## Abstract

The coexistence of multiple actin crosslinking proteins in cells suggests that crosslinkers with distinct binding dynamics may cooperate to organize F-actin networks that support adhesion, motility, and division. Here, we examine how crosslinker identity and lifetime regulate actin-network structure and mechanics using a reconstituted system that combines α -actinin, a native dynamic actin crosslinker, with biotin–NeutrAvidin, a non-native model of long-lived, effectively persistent crosslinking. Using confocal fluorescence microscopy and optical-tweezers microrheology, we compared networks formed with α -actinin alone, biotin–NeutrAvidin alone, or a representative equimolar 50:50 mixture of the two crosslinking schemes at fixed total crosslinker-to-actin ratio, R . Mixed crosslinking produced the strongest mesoscale structural heterogeneity at high R , yielding larger characteristic structural features than either pure-crosslinker network. However, this structural coarsening did not translate into uniformly enhanced linear viscoelasticity: mixed networks generally exhibited moduli and viscosities intermediate between those of pure α -actinin and biotin–NeutrAvidin networks. Nonlinear microrheology further revealed that crosslinker identity and lifetime regulate force buildup, strain stiffening, and stress relaxation in an observable-dependent manner. Thus, the representative mixed-crosslinker network did not simply generate the strongest or most solid-like actin network. Instead, its effects were selective: it enhanced mesoscale heterogeneity at high R , produced intermediate linear and total nonlinear force responses, and gave rise to distinct nonlinear stiffening and relaxation behavior. These results demonstrate that cooperation between dynamic and persistent crosslinks is deformation-regime dependent, providing a mechanism for tuning actin-network architecture and mechanics without uniformly increasing rigidity.

## 1 Introduction

The mechanical properties of F-actin networks are central to cellular structure, force transmission, motility, division, and mechanosensing. These properties are not determined by actin filaments alone, but by the actin-binding proteins that organize filaments into networks, bundles, and higher-order assemblies. Crosslinking proteins are especially important because they regulate filament connectivity, network remodeling, stress storage, and stress relaxation. As a result, changing crosslinker identity, concentration, or binding lifetime can strongly alter both the architecture and mechanics of actin-based materials (Gardel et al., 2008; Blanchoin et al., 2014; Svitkina, 2018; Cooper, 2000; Chang et al., 2014).

A key example is α -actinin, a native actin-binding protein that dynamically crosslinks F-actin and can organize filaments into either relatively isotropic networks or bundled structures, depending on concentration, binding dynamics, and filament organization (Gardel et al., 2010; Schwarz and Gardel, 2012). In reconstituted actin systems, reported α -actinin dissociation rates from F-actin range from approximately 0.2 to $5.2\;s^{-1}$ , corresponding to characteristic bond lifetimes from fractions of a second to several seconds, depending on isoform, temperature, and experimental conditions (Wachsstock et al., 1994; Xu et al., 1998). Live-cell FRAP measurements similarly show rapid α -actinin turnover at the actin cortex, with recovery occurring on the order of seconds (Fritzsche et al., 2013; Hosseini et al., 2020). This dynamic exchange allows α -actinin-crosslinked networks to remodel and relax stress while still supporting elastic stress transmission over short and intermediate timescales.

The structural and mechanical effects of α -actinin are therefore concentration- and timescale-dependent. At relatively low concentrations, α -actinin can increase filament connectivity while maintaining a more isotropic mesh, thereby enhancing the linear viscoelastic response of the network. At higher concentrations, its bundling activity becomes more pronounced, promoting coarser architectures that can resist deformation more strongly (Stricker et al., 2010; Wen and Janmey, 2011; Gardel et al., 2010; Schwarz and Gardel, 2012). Thus, α -actinin regulates actin mechanics not simply by increasing the number of filament connections, but by coupling reversible binding, filament rearrangement, and bundle formation.

Biotin–NeutrAvidin provides a useful contrasting crosslinking scheme for testing how persistent connectivity affects actin-network mechanics. NeutrAvidin is not a native actin-binding protein; rather, it binds biotinylated actin filaments to create non-native, long-lived crosslinks in reconstituted networks. Compared with α -actinin, biotin–NeutrAvidin linkages are effectively persistent on the timescales of typical microrheology measurements and therefore impose more static constraints on filament motion. This contrast makes the biotin–NeutrAvidin system useful for separating the effects of dynamic, reversible crosslinking from those of long-lived crosslink-mediated connectivity.

Previous studies of mixed actin-binding proteins show that different crosslinkers can cooperate to alter actin-network mechanics, but they also demonstrate that the outcome depends on the molecular properties of the proteins involved and on the mechanical observable being measured. For example, combined α -actinin and fascin produced a larger mechanical response than either protein alone, supporting the idea that crosslinkers with distinct actin-organizing functions can act cooperatively (Tseng et al., 2005). In reconstituted networks, α -actinin and filamin were shown to cooperatively enhance elastic stiffness, with effects reflected not only in the steady-state elastic modulus, but also in the frequency dependence of $G^{\prime }\left(\omega \right)$ , the scaling exponent in $G^{\prime }\left(\omega \right)∼\omega^{\alpha }$ , the phase angle $\delta =\tan^{-1}\left(G^{\prime \prime }/G^{\prime }\right)$ , and nonlinear shear stiffening and softening under increasing deformation amplitude (Esue et al., 2009). Other crosslinker systems similarly show that structural transitions, bundling, and crosslinker concentration can alter $G^{\prime }\left(\omega \right)$ , $G^{\prime \prime }\left(\omega \right)$ , plateau modulus, and nonlinear strain response in ways that are not always monotonic (Luan et al., 2008; Lieleg and Bausch, 2007; Grooman et al., 2012). These studies motivate a broader question: does combining crosslinkers with different binding lifetimes necessarily produce a stronger actin network, or does the outcome depend on which structural or mechanical observable is measured?

This question is especially important in nonlinear microrheology, where the measured response depends not only on crosslinker identity but also on the length and rate scales probed by the moving bead. Prior work showed that actin-network mechanics depends on how the probe size, imposed displacement, mesh size, tube diameter, entanglement length, and crosslink spacing compare with one another (Falzone et al., 2015; Gurmessa et al., 2016; Gurmessa et al., 2017). In particular, crosslinker-mediated connectivity is expected to dominate when the average distance between crosslinks becomes smaller than the entanglement length. These scaling ideas help distinguish whether nonlinear resistance arises mainly from actin entanglements, crosslink constraints, or collective deformation of the mesoscale network.

Here, we address these questions using reconstituted F-actin networks crosslinked by α -actinin, biotin–NeutrAvidin, or a 50:50 mixture of the two crosslinking schemes at a fixed total crosslinker-to-actin ratio, R . This design allows us to compare a native dynamic crosslinker with a non-native persistent crosslinking linkage, both individually and in combination. By combining confocal fluorescence microscopy with optical-tweezers microrheology, we examine how crosslinker identity and lifetime regulate mesoscale architecture, linear viscoelasticity, nonlinear stiffening, and post-strain stress relaxation.

## 2 Materials and methods

Proteins: Lyophilized unlabeled (A) and biotinylated (BA) rabbit skeletal muscle globular actin (G-actin) (Cytoskeleton, Inc., AKL99, AB07) were resuspended to concentrations of 46 μ M (2 mg/mL ) for A and 23.8 μ M (1 mg/mL ) for BA in Ca Buffer G [2 mM Tris, pH 8.0, 0.2 mM ATP, 0.5 mM DTT, 0.1 mM $CaCl_{2}$ ] and stored at −80° C. α -actinin and Acti-Stain 555 Phalloidin (Cytoskeleton, Inc.) were reconstituted to 10 μ M (1 mg/mL ) and 14 μ M (2.5 mg/mL ), in deionized water and methanol, respectively. α -actinin was stored at −80° C in buffer [4 mM Tris-HCl, pH 7.6, 4 mM NaCl, 20 μM EDTA, 1% (w/v) sucrose, 0.2% (w/v) dextran], while phalloidin was stored at −20° C in 100% methanol.

Network Preparation: Crosslinked F-actin networks were prepared at a final actin concentration of 5.8 μM $\left(c_{a}≃0.244\;mg/mL\right)$ in F-buffer [10 mM imidazole, pH 7.0, 50 mM KCl, 1 mM $MgCl_{2}$ , 1 mM EGTA, and 0.2 mM ATP]. Networks contained unlabeled G-actin, biotinylated actin, Acti-Stain 555 phalloidin, oxygen-scavenging reagents, crosslinkers, and trace amounts of polystyrene microspheres for optical-tweezers microrheology. Phalloidin was added at a phalloidin:actin molar ratio of $R_{p}=0.02$ . Oxygen-scavenging reagents consisted of 4.5 mg/mL glucose, 0.005% β -mercaptoethanol, 43 μg/mL glucose oxidase, and 7 μg/mL catalase.

For all network conditions, the total actin concentration was held fixed at 5.8 μM . In the biotin–NeutrAvidin-only and 50:50 mixed networks, biotinylated actin was included as part of this fixed total actin concentration, such that ${\left[actin\right]}_{\text{total}}=\left[non-biotinylated\;actin\right]+\left[biotinylated\;actin\right]=5.8\;\mu M$ . The α -actinin-only networks contained no biotinylated actin and therefore consisted entirely of non-biotinylated actin at the same total concentration of 5.8 μM . Thus, biotinylated actin replaced an equivalent fraction of non-biotinylated actin in the NeutrAvidin-containing samples rather than increasing the total actin concentration. For biotin–NeutrAvidin-crosslinked networks, biotinylated actin was pre-complexed with NeutrAvidin at a twofold molar excess of biotinylated actin to NeutrAvidin (BA:NA = 2:1). The total crosslinker-to-actin molar ratio, R , was varied as 0, 0.1, 0.2, and 0.3. For mixed-crosslinker networks, the total R was held fixed while the crosslinking contribution was divided equally between α -actinin and biotin–NeutrAvidin. Thus, the 50:50 networks contained equal contributions from the dynamic α -actinin crosslinking mechanism and the long-lived biotin–NeutrAvidin linkage at the same total crosslinker-to-actin ratio. Specifically, for the 50:50 mixed networks, the total crosslinker-to-actin ratio was defined as $R=R_{\alpha }+R_{\text{NA}}$ , where $R_{\alpha }=\left[\alpha -\text{actinin}\right]/\left[actin\right]$ and $R_{\text{NA}}=\left[NeutrAvidin\right]/\left[actin\right]$ . Thus, for a given total R , the mixed networks contained $R_{\alpha }=R/2$ and $R_{\text{NA}}=R/2$ , with biotinylated actin included at a twofold molar excess relative to NeutrAvidin. Throughout the manuscript, R is interpreted as a nominal preparation variable, not as a direct measurement of mechanically active crosslink density or effective network connectivity. The definitions of R for each crosslinking condition are summarized in Table 1. This distinction is important because α -actinin and biotin–NeutrAvidin differ in binding geometry, apparent valency, binding lifetime, and dependence on biotinylated actin.

**TABLE 1:** Definition of the nominal total crosslinker-to-actin molar ratio, R , for each network condition, with an example for networks prepared at total R=0.2 .

Network type	Nominal R used in this work	Component ratios	For example, at R=0.2
α -actinin only	R=[α-actinin]/[actin]	All nominal crosslinker is α -actinin	$R_{\alpha }=0.2$
Biotin–NeutrAvidin only	R=[NeutrAvidin]/[actin]	All nominal crosslinker is NeutrAvidin	$R_{\text{NA}}=0.2$
50:50 mixed	$R=R_{\alpha }+R_{\text{NA}}$	$R_{\alpha }=R/2$ , $R_{\text{NA}}=R/2$	$R_{\alpha }=0.1$ , $R_{\text{NA}}=0.1$

This table is included to clarify that equal nominal R does not imply equal effective crosslink density.

Trace amounts of 4.2 μm polystyrene microspheres (Bangs Laboratories) were added before polymerization for microrheology measurements. The solutions were flowed into sample chambers constructed from glass slides and coverslips separated by approximately 100 μm , sealed with epoxy, and allowed to polymerize and crosslink for 1 h at room temperature. Network mesh sizes were estimated from the actin concentration using established scaling relations, $\xi_{m}\approx 0.3c_{a}^{-1/2}\approx 0.61\;\mu m$ , where $c_{a}$ is the actin concentration in mg/mL (Gardel et al., 2003; Schmidt et al., 1989).

Microscopy: Network architecture was visualized using a Leica TCS SP5 confocal microscope equipped with a 63×1.4 NA oil-immersion objective and a 561 nm excitation laser. Three-dimensional image stacks comprising 100 optical sections ( 512×512 pixels) were acquired at 0.5 μm z -intervals, yielding a lateral field of view of approximately 246.5 μm×246.5 μm and a total z -range of 50 μm . These structural datasets were directly associated with microrheology measurements, ensuring that the mechanical characterization corresponded to the imaged networks (Falzone and Robertson-Anderson, 2015; Gurmessa et al., 2017). For time-stack imaging, confocal images were acquired at a fixed focal plane over time under the same excitation and detection settings. Each time series contained 139 frames acquired at 1 s intervals (1 frame/s) , corresponding to approximately 139 s of imaging, or 138 s elapsed from the first to last frame. The resulting time series were projected using mean-intensity projections and analyzed by spatial image autocorrelation in the same manner as the z -stack projections.

To quantitatively assess network morphology, we performed spatial image autocorrelation (SIA) analysis using a custom Python script (Robertson and George, 2012). SIA computes the spatial correlation function, g(r) , of image intensity values, I(r) , for pixel pairs separated by a distance r . For each image, g(r) was obtained by computing the fast Fourier transform (FFT), multiplying it by its complex conjugate, applying the inverse FFT, and normalizing by the squared mean intensity:

$$g\left(r\right)=\frac{F^{-1}\left(|F\left(I\left(r\right)\right){|}^{2}\right)}{{\left\langle I\left(r\right)\right\rangle }^{2}},$$

where F and $F^{-1}$ denote the forward and inverse Fourier transforms, respectively. The structural correlation length, $\xi_{\text{SIA}}$ , was extracted by fitting the short-distance decay of g(r) to $g\left(r\right)=g_{0}\exp\left(-\frac{r}{\xi_{\text{SIA}}}\right)$ , where $g_{0}$ is the fitted autocorrelation amplitude.

Microrheology: Optical tweezers microrheology measurements were performed using a custom-built system based on an IX73 fluorescence microscope (Olympus) equipped with a 1064 nm Nd:YAG fiber laser (BKtel, RPMC Lasers). The laser was focused through a 60× 1.42 NA oil-immersion objective (UPLXAPO60XO, Olympus). A position-sensing detector (PSM2-10Q PSD/OT-301, ON-TRAK Photonics) recorded laser deflections proportional to forces on trapped probes. Calibration of trap stiffness was performed using Stokes drag in water (Williams, 2002) and passive equipartition methods (Brau et al., 2007).

For passive, linear microrheology measurements, embedded probe beads were optically trapped but not actively displaced. For each sample condition, measurements were collected from independent beads at different locations within the network to obtain an ensemble-averaged response. Each bead was held in the optical trap for 130 s , and bead-position fluctuations were recorded at 20 kHz using the position-sensing detector. Because the probe bead was not externally displaced, its motion arose from thermally driven fluctuations of the surrounding network. These small-amplitude fluctuations probe the near-equilibrium, linear viscoelastic response of the material without appreciably deforming the network (Tassieri et al., 2012; Pinchiaroli et al., 2024).

The recorded bead-position fluctuations were converted into the frequency-dependent storage and loss moduli, $G^{\prime }\left(\omega \right)$ and $G^{\prime \prime }\left(\omega \right)$ , using a generalized Stokes–Einstein relation approach based on the normalized mean-squared displacement (NMSD). The NMSD was calculated as

$$\Pi \left(\tau \right)=\frac{\langle \Delta r^{2}\left(\tau \right)\rangle }{2\langle r^{2}\rangle },$$

where τ is the lag time, $\left\langle \Delta r^{2}\left(\tau \right)\right\rangle$ is the mean-squared displacement of the trapped bead, and $\left\langle r^{2}\right\rangle$ is the mean-squared bead displacement about the trap center. The Fourier transform of the NMSD, $\hat{\Pi }\left(\omega \right)$ , was evaluated using the discrete transform expression

$$-\omega^{2}\hat{\Pi }\left(\omega \right)=\frac{\Pi \left(\tau_{1}\right)}{\tau_{1}}\left(1-e^{-i\omega \tau_{1}}\right)+{\dot{\Pi }}_{\infty }e^{-i\omega \tau_{N}}+\overset{N}{\underset{k=2}{\sum }}\frac{\Pi_{k}-\Pi_{k-1}}{\tau_{k}-\tau_{k-1}}\left(e^{-i\omega \tau_{k-1}}-e^{-i\omega \tau_{k}}\right),$$

where ${\dot{\Pi }}_{\infty }$ is the long-time slope of Π(τ) , $\tau_{N}$ is the final lag time, and $\tau_{1}$ and $\tau_{N}$ correspond to the first and last points of the oversampled NMSD, respectively (Tassieri et al., 2012; Pinchiaroli et al., 2024). To improve numerical stability over the measured lag-time range, Π(τ) was oversampled using the piecewise cubic Hermite interpolating polynomial, PCHIP, function in MATLAB prior to Fourier transformation.

The complex shear modulus was then calculated from

$$G^{*}\left(\omega \right)=G^{\prime }\left(\omega \right)+iG^{\prime \prime }\left(\omega \right)=\frac{\kappa }{6\pi a}\left[\frac{1}{i\omega \hat{\Pi }\left(\omega \right)}-1\right],$$

where κ is the optical trap stiffness and a is the bead radius. The storage modulus, $G^{\prime }\left(\omega \right)$ , quantifies the elastic or solid-like response of the network, whereas the loss modulus, $G^{\prime \prime }\left(\omega \right)$ , quantifies the viscous or fluid-like response. The magnitude of the complex viscosity was calculated as $\eta_{\text{eff}}\left(\omega \right)=\frac{{\left[G^{\prime }{\left(\omega \right)}^{2}+G^{\prime \prime }{\left(\omega \right)}^{2}\right]}^{1/2}}{\omega }.$

Following passive linear microrheology, nonlinear microrheology measurements were performed by actively displacing embedded probe beads through the actin networks. Probes were translated 10 μm at a constant speed of 5 μm/s using a piezoelectric stage (PDQ-250, Mad City Labs). The imposed bead speed corresponds to a characteristic strain rate $\dot{\gamma }\approx 5.0\;s^{-1}$ , which exceeds the reported nonlinear crossover rate ${\dot{\gamma }}_{c}∼3\;s^{-1}$ for entangled actin networks (Falzone et al., 2015), while remaining sufficiently slow to prevent bead ejection from the optical trap (Gurmessa et al., 2016; Gurmessa et al., 2017). Force data were acquired at 20 kHz over three sequential phases: equilibration before bead displacement (5 s) , active strain during stage translation (2 s) , and relaxation after the imposed displacement (15 s) .

Data Analysis: Each crosslinker condition and total crosslinker-to-actin ratio, R , was prepared independently in two separate sample preparations. For linear microrheology, 20 bead trajectories were analyzed from each preparation, yielding N=40 bead-level measurements per condition. For nonlinear microrheology, 20 bead-pull trials were performed for each preparation, yielding N=40 trial-level measurements per condition. Nonlinear force-response curves were obtained using unique probe beads positioned >100 μm apart to minimize local spatial correlations between measurements. Measurements obtained from multiple beads or bead-pull trials within the same preparation were treated as technical replicates nested within independent sample preparations, rather than as fully independent sample preparations. Error bars in force plots represent the standard error across bead-pull trials. Repeated experiments confirmed that the observed trends were reproducible across independently prepared samples, as shown in Supplementary Figures S3 and S4. Custom LabVIEW software was used for instrument control and data acquisition, and all subsequent analyses were performed in MATLAB.

## 3 Results

To determine how the crosslinking mechanism regulates F-actin network structure and mechanics, we combined confocal fluorescence microscopy with passive and active optical-tweezers microrheology. Networks were prepared at fixed actin concentration and compared across three crosslinking conditions: pure α -actinin, pure biotin–NeutrAvidin, and a 50:50 mixture of the two crosslinking schemes at fixed total crosslinker-to-actin ratio, R . Because only one mixed ratio was examined, this design tests whether the equimolar mixed condition differs from the two single-crosslinker limits; it does not establish how mechanics varies across all α -actinin:NeutrAvidin mixing ratios. This design allowed us to test whether combining a native dynamic crosslinker with a long-lived biotin–NeutrAvidin linkage produces a simple intermediate response, a uniformly enhanced mechanical response, or an observable-dependent outcome. Below, we present the results by moving from structure to mechanics: first examining how crosslinker composition changes mesoscale network architecture, then quantifying the small-amplitude linear viscoelastic response, and finally testing how the networks respond to large local deformation through force buildup, differential stiffening, and post-strain relaxation.

### 3.1 Mixed crosslinking produces the strongest mesoscale structural heterogeneity at high crosslinker concentration

**FIGURE 1:**
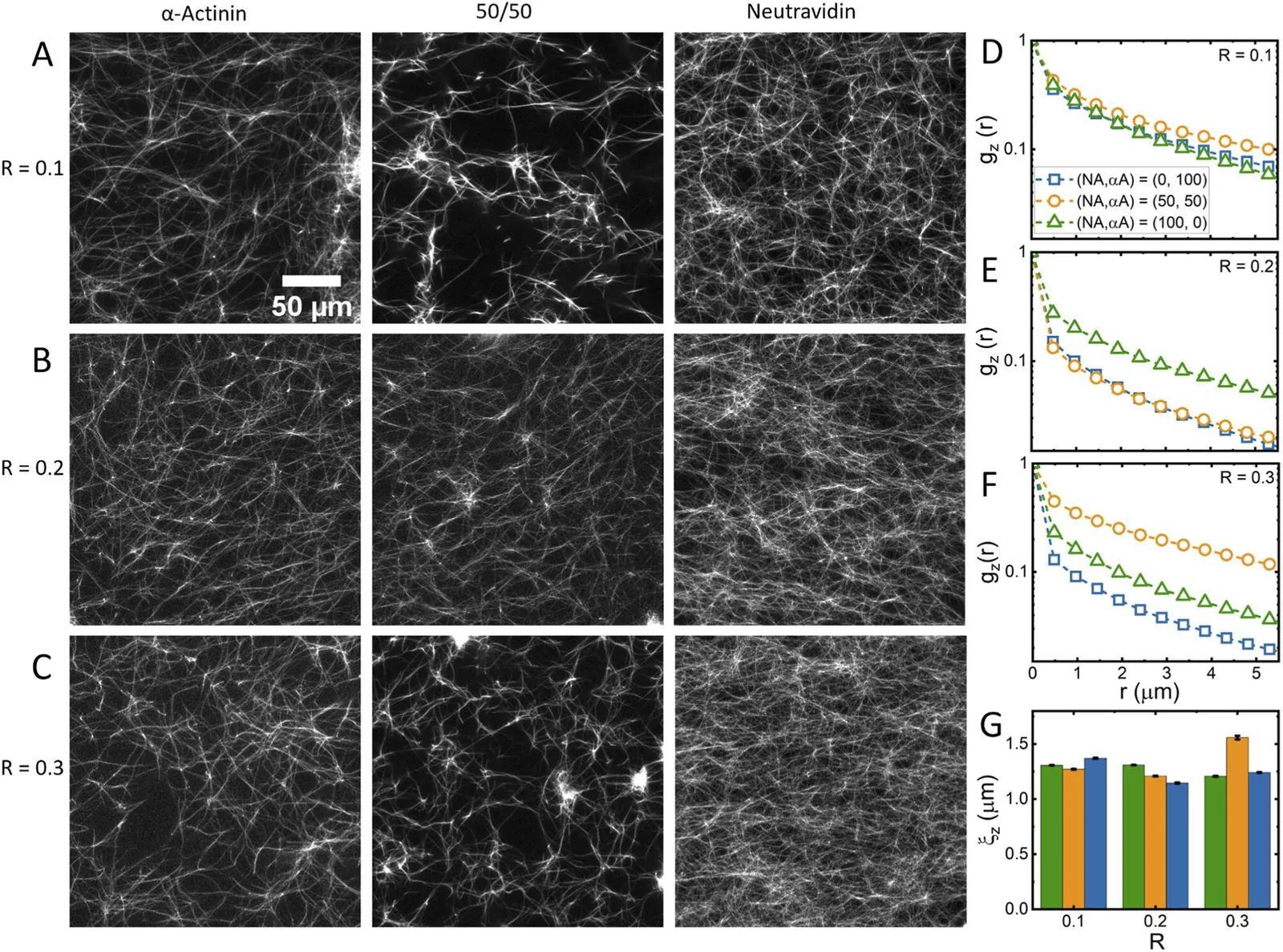
Three-dimensional confocal imaging reveals composition-dependent mesoscale architecture in crosslinked F-actin networks. **(A–C)** Mean-intensity projections of confocal z -stacks of phalloidin-labeled F-actin networks formed with pure α -actinin, a 50:50 α -actinin/biotin–NeutrAvidin mixture, or pure biotin–NeutrAvidin at total crosslinker-to-actin ratios R=0.1 , 0.2, and 0.3. Scale bar: 50 μm . Mixed 50:50 networks appear more spatially heterogeneous than the pure-crosslinker networks, particularly at R=0.3 , where bright bundled regions coexist with larger dark voids. **(D–F)** Spatial autocorrelation functions, $g_{z}\left(r\right)$ , computed from the projected z -stack images for each R . The legend in panel D applies to panels D–G. Slower decay of $g_{z}\left(r\right)$ indicates larger correlated structural features. **(G)** Characteristic structural correlation length, $\xi_{z}$ , obtained from exponential fits to the autocorrelation functions. The largest structural length scale occurs for the 50:50 network at R=0.3 , indicating that mixed transient and effectively persistent crosslinking produces the most pronounced mesoscale coarsening among the conditions tested. Numerical fit values for the extracted correlation lengths, together with the corresponding fit ranges, fit uncertainties, and corresponding goodness-of-fit values $\left(R^{2}\right)$ , have been added to Supplementary Table S3. The table reports results obtained using both the short-range fitting window (0.481– 5 μm ) and the extended-range fitting window (0.481– 15 μm ), allowing direct comparison of the short- and long-range autocorrelation fits. The r=0 point was excluded from the fits because it represents the trivial self-correlation value, g(0)=1 , and is commonly followed by a sharp initial decrease that may be influenced by pixel-scale effects, finite image resolution, or other sub-resolution features.

We first examined whether crosslinker composition altered the mesoscale architecture of the actin networks. Confocal z -stack projections of phalloidin-labeled F-actin networks revealed composition-dependent differences in filament organization (Figure 1). At R=0.1 and R=0.3 , the 50:50 projections showed more evident large-scale spatial segregation into bright filament-rich regions and dark filament-poor regions than the pure-crosslinker projections. This spatial organization was less apparent at R=0.2 . The distinction was clearest at R=0.3 , where the mixed network exhibited the largest apparent voids and the coarsest spatial organization. These qualitative observations refer to the spatial size and arrangement of the network features rather than to the overall magnitude of pixel-intensity variation.

To quantify these visual differences, we performed spatial image autocorrelation analysis on z -stack and time-stack confocal images. The normalized autocorrelation function, g(r) , measures how strongly image intensities remain correlated over a distance r . Because the curves were normalized, differences among conditions were interpreted from the decay length and shape of g(r) , rather than from the initial amplitude. A slower decay of g(r) indicates larger characteristic structural features, whereas a faster decay indicates smaller or more finely distributed network features.

The autocorrelation curves exhibited an approximately exponential decay over short distances, so the initial decay region, up to ∼5 μm , was fit to g(r) as described in Methods. The fit was restricted to this initial decay region because the long-distance tails of g(r) were not always well described by a single exponential and can be influenced by image-level background variations or flat-field noise, as noted in prior SIA analyses of cytoskeletal networks (Gurmessa et al., 2021; Lee et al., 2021; Sheung et al., 2021). In this context, $\xi_{\text{SIA}}$ reports the typical length scale over which fluorescence intensity remains spatially correlated. Larger $\xi_{\text{SIA}}$ values indicate coarser network organization, such as thicker bundles, larger pores, or stronger spatial heterogeneity. Because $\xi_{\text{SIA}}$ is extracted from the short-distance decay of the autocorrelation function, it should be interpreted as a local-to-mesoscale correlation length rather than as a direct measure of the largest voids or macropores. As a complementary measure of fluorescence-intensity heterogeneity, we calculated the intensity coefficient of variation, ${CV}_{I}$ , from both z -series and time-series confocal images (Supplementary Figure S5), according to ${CV}_{I}=\frac{\sigma_{I}}{\left\langle I\right\rangle }$ , where ⟨I⟩ and $\sigma_{I}$ are the mean and standard deviation, respectively, of the background-subtracted fluorescence intensities within the analyzed region of interest. Higher ${CV}_{I}$ values indicate a broader distribution of fluorescence intensities and therefore greater image-level intensity heterogeneity. Biotin–NeutrAvidin-only networks generally exhibited the lowest ${CV}_{I}$ values across matched R , whereas networks containing α -actinin, either alone or in 50:50 mixtures, exhibited greater intensity variation. This increased variation may reflect differences in local filament density, bundle brightness, or filament-depleted regions; however, ${CV}_{I}$ does not quantify the characteristic size, spacing, or spatial arrangement of these features.

The z -stack autocorrelation curves were relatively similar among the three crosslinker compositions at R=0.1 and R=0.2 , but separated more clearly at R=0.3 (Figures 1D–F). At this highest crosslinker ratio, the 50:50 network exhibited the slowest-decaying autocorrelation curve and the largest extracted structural correlation length, $\xi_{z}$ , indicating the largest characteristic structural features among the three compositions (Figure 1G). Thus, while ${CV}_{I}$ reports the magnitude of fluorescence-intensity variation, $\xi_{z}$ and $\xi_{t}$ report the characteristic spatial scale over which those intensity variations are organized. Consequently, a network may exhibit a high ${CV}_{I}$ without a corresponding increase in correlation length, as observed at R=0.2 , or a large correlation length without exhibiting the highest overall intensity variation, as observed for the 50:50 network at R=0.3 .

**FIGURE 2:**
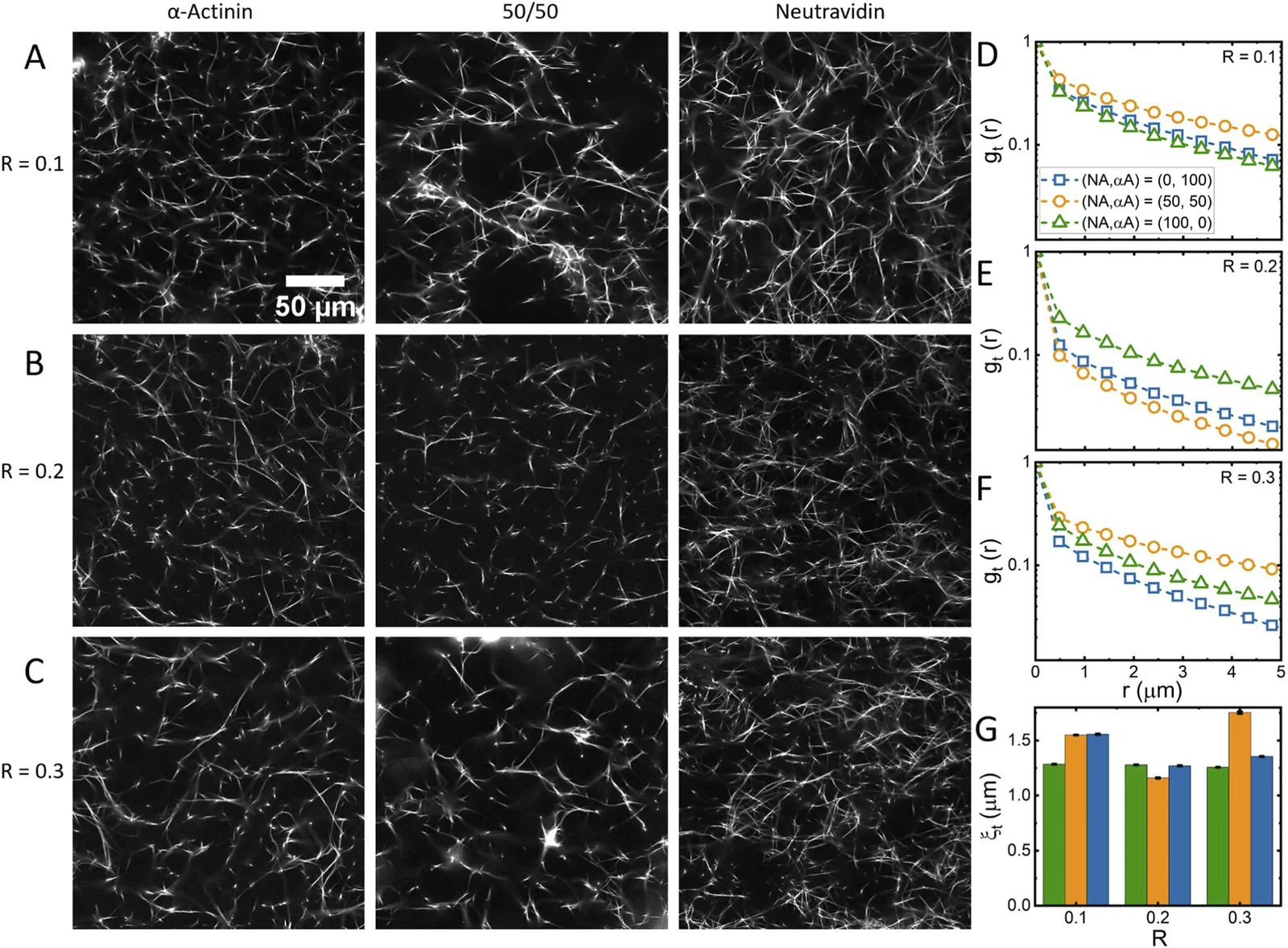
Time-stack confocal projections confirm crosslinker-dependent actin-network heterogeneity. **(A–C)** Mean-intensity projections of confocal time stacks acquired at fixed sample positions for phalloidin-labeled F-actin networks crosslinked with pure α -actinin, a 50:50 α -actinin/biotin–NeutrAvidin mixture, or pure biotin–NeutrAvidin at R=0.1 , 0.2, and 0.3. Scale bar: 50 μm . The time-stack projections reinforce the structural trends observed in the z -stack images, with mixed networks exhibiting prominent bright bundles and larger voids, most clearly at R=0.3 . **(D–F)** Spatial autocorrelation functions, $g_{t}\left(r\right)$ , calculated from the time-stack projections. **(G)** Correlation length, $\xi_{t}$ , extracted from exponential fits to $g_{t}\left(r\right)$ . The 50:50 network exhibits the largest $\xi_{t}$ at R=0.3 , indicating that the structural signature of mixed crosslinking is reproducible across both z -stack and time-stack imaging modes. The legend in panel D applies to panels D–G. Numerical values of the extracted correlation lengths, together with the corresponding fit ranges, fit uncertainties, and goodness-of-fit values $\left(R^{2}\right)$ , are reported in Supplementary Table S3. The table includes results obtained using both a short-range fitting window (0.481– 5 μm ) and an extended-range fitting window (0.481– 15 μm ), enabling direct comparison of the extracted correlation lengths across the two fitting regimes. The r=0 point was excluded from the fits because it represents the trivial self-correlation value, g(0)=1 , and is commonly followed by a sharp initial decrease that may be influenced by pixel-scale effects, finite image resolution, or other sub-resolution features.

Time-stack projections supported the same structural trend (Figure 2). Consistent with the z -stack images, mixed 50:50 networks appeared more heterogeneous than the pure-crosslinker networks, especially at R=0.3 . Spatial autocorrelation analysis of the time-stack projections again showed that the 50:50 network had the largest structural correlation length at R=0.3 . Thus, the main structural effect of mixing α -actinin and biotin–NeutrAvidin was not a uniform increase in network density or a monotonic change across all concentrations. Instead, the clearest structural distinction emerged at high total crosslinker concentration, where mixed dynamic and persistent crosslinking produced the coarsest mesoscale architecture.

### 3.2 Linear microrheology shows that α -actinin controls modulus magnitude and concentration sensitivity

We next asked how these structural differences were reflected in the linear viscoelastic response. Passive optical-tweezers microrheology was performed before active bead displacement. Thermal bead fluctuations were converted into a normalized mean-squared displacement and used to determine the complex modulus, $G^{*}\left(\omega \right)=G^{\prime }\left(\omega \right)+iG^{\prime \prime }\left(\omega \right)$ , following established optical-tweezers microrheology analysis methods (see Materials and Methods for details). The storage modulus, $G^{\prime }\left(\omega \right)$ , reports the elastic, energy-storing component of the response, whereas the loss modulus, $G^{\prime \prime }\left(\omega \right)$ , reports the viscous, energy-dissipating component (Tassieri et al., 2012; Pinchiaroli et al., 2024).

**FIGURE 3:**
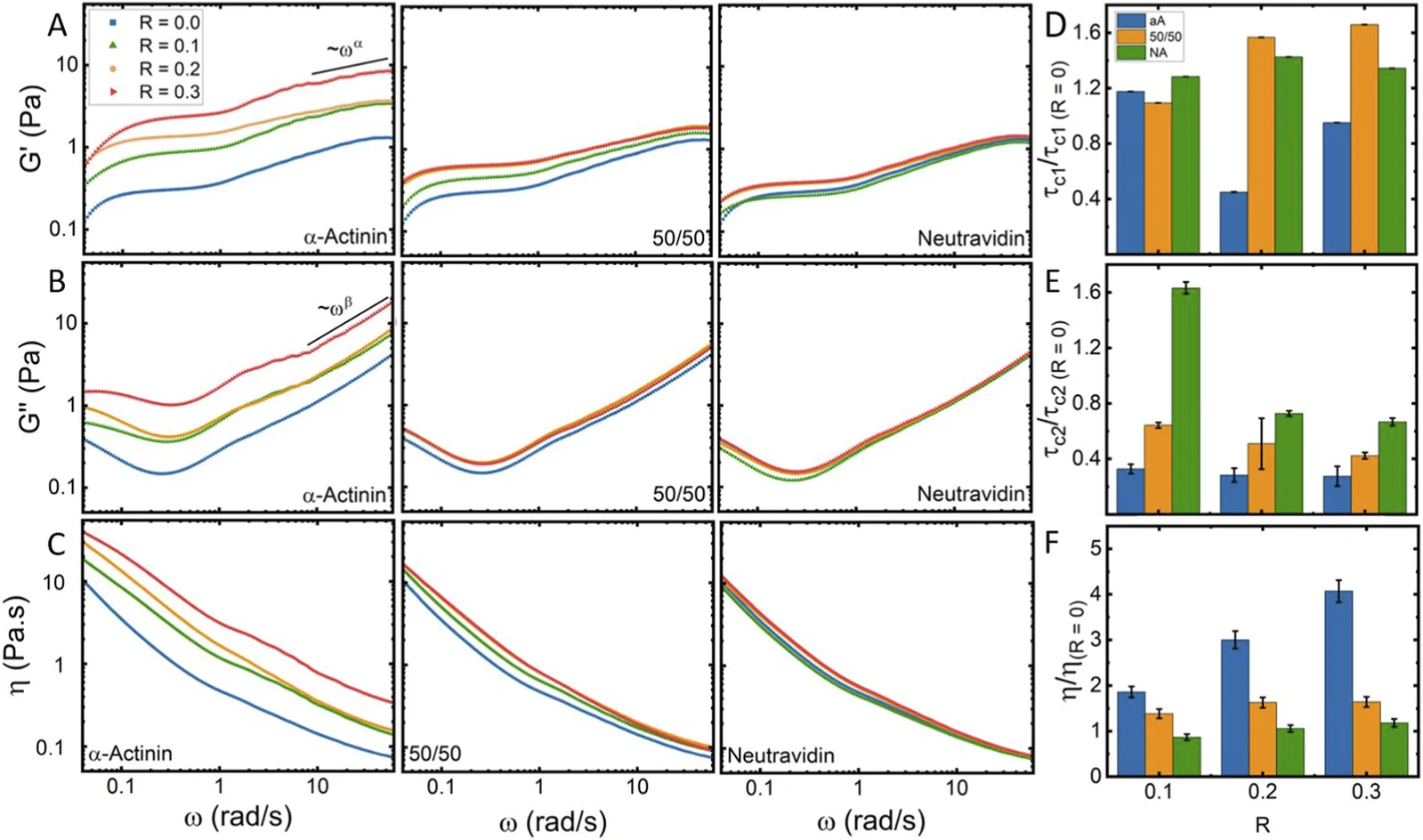
Linear viscoelastic response of crosslinked F-actin networks depends on crosslinker identity and concentration **(A)** Storage modulus, $G^{\prime }\left(\omega \right)$ , measured from passive optical-tweezers microrheology for networks crosslinked with pure α -actinin, 50:50 α -actinin/biotin–NeutrAvidin, or pure biotin–NeutrAvidin **(B)** Loss modulus, $G^{\prime \prime }\left(\omega \right)$ , for the same conditions **(C)** Effective frequency-dependent viscosity, $\eta_{\text{eff}}\left(\omega \right)=|G^{*}\left(\omega \right)|/\omega$ . In the α -actinin networks, $G^{\prime }$ , $G^{\prime \prime }$ , and $\eta_{\text{eff}}$ show the strongest separation with increasing R , indicating pronounced concentration sensitivity. Biotin–NeutrAvidin-crosslinked networks show more clustered moduli, indicating weaker concentration dependence in the linear regime, while the 50:50 networks generally fall between the two pure-crosslinker limits **(D,E)** Crossover-time summaries extracted from the frequency-dependent moduli **(F)** Effective viscosity, $\eta_{\text{eff}}$ , normalized to the uncrosslinked control. In panels D–F, blue denotes pure α -actinin networks, orange denotes 50:50 α -actinin/biotin–NeutrAvidin networks, and green denotes pure biotin–NeutrAvidin networks. Panels D–F are normalized by the corresponding uncrosslinked R=0 actin-network values; therefore, the R=0 reference corresponds to a normalized value of 1.

Across all crosslinker compositions, $G^{\prime }\left(\omega \right)$ increased with frequency, consistent with frequency-dependent elasticity in semiflexible polymer networks (Figure 3A). However, the sensitivity of $G^{\prime }\left(\omega \right)$ to total crosslinker-to-actin ratio, R , depended strongly on crosslinker identity. Pure α -actinin networks showed the largest separation among R values, with $G^{\prime }\left(\omega \right)$ increasing substantially as R increased. In contrast, the biotin–NeutrAvidin curves were more closely clustered, indicating weaker concentration sensitivity over the range tested. The 50:50 networks generally fell between the two pure-crosslinker limits, showing that mixed crosslinking did not simply exceed both pure-crosslinker conditions in linear elasticity.

The loss modulus, $G^{\prime \prime }\left(\omega \right)$ , showed a similar but not identical trend (Figure 3B). In pure α -actinin networks, $G^{\prime \prime }\left(\omega \right)$ increased strongly with R , indicating that α -actinin increased both elastic storage and viscous dissipation in the linear regime. The 50:50 condition again showed intermediate behavior, whereas biotin–NeutrAvidin-crosslinked networks exhibited more weakly separated $G^{\prime \prime }\left(\omega \right)$ curves across R . Thus, both moduli showed that α -actinin had the strongest effect on the magnitude and concentration dependence of the small-amplitude viscoelastic response.

Because the frequency-dependent effective viscosity, $\eta_{\text{eff}}\left(\omega \right)=|G^{*}\left(\omega \right)|/\omega$ , depends on the combined magnitude of $G^{\prime }\left(\omega \right)$ and $G^{\prime \prime }\left(\omega \right)$ , it provides a compact summary of the linear viscoelastic response (Figures 3C,F). Pure α -actinin networks exhibited the largest viscosity at every R , and the separation from the other crosslinker conditions increased with concentration. At R=0.1 , the viscosity of the α -actinin network was roughly 40% higher than the 50:50 network and nearly two-fold higher than the biotin–NeutrAvidin network. By R=0.3 , the α -actinin viscosity was approximately three-fold higher than the 50:50 network and more than four-fold higher than biotin–NeutrAvidin. Thus, among the linear quantities, effective viscosity showed one of the clearest crosslinker-identity dependences. This frequency-dependent viscosity should be interpreted as an apparent linear microrheological measure of resistance that integrates contributions from both elastic storage and viscous loss. For α -actinin-containing networks, the measured frequency window overlaps reported molecular exchange rates, $k_{\text{off}}∼0.2$ – $5.2\;s^{-1}$ , suggesting that changes in $\eta_{\text{eff}}\left(\omega \right)$ , $G^{\prime }$ , and $G^{\prime \prime }$ may reflect a combination of network architecture and reversible crosslinker exchange. To clarify this interpretation, red vertical arrows in Supplementary Figure S1 mark the approximate kinetic window associated with these reported α -actinin unbinding rates. These markers are intended as visual guides to the frequency range over which crosslinker exchange may influence the linear viscoelastic response, rather than as precise crossover frequencies. In contrast, biotin–NeutrAvidin linkages are expected to remain effectively persistent over this frequency window.

We also examined the frequency-dependent shape of the spectra by extracting crossover times and high-frequency power-law exponents. Crossover points were identified where $G^{\prime }\left(\omega \right)=G^{\prime \prime }\left(\omega \right)$ , and the corresponding crossover frequencies were converted to characteristic timescales, $\tau_{c1}=2\pi /\omega_{c1}$ and $\tau_{c2}=2\pi /\omega_{c2}$ (Figures 3D,E; see also Supplementary Figure S1). As frequency increases, the first crossover marks entry into an elasticity-dominated regime, where $G^{\prime }\left(\omega \right)>G^{\prime \prime }\left(\omega \right)$ , whereas the second crossover marks exit from this regime and return to viscosity-dominated behavior at higher frequencies. Because these quantities are normalized by their corresponding R=0 values, values above or below unity indicate shifts relative to the uncrosslinked reference. To assess crosslinker identity, however, we compared the three crosslinking conditions at fixed R .

The crossover times showed strong crosslinker-identity dependence, but the two crossovers followed different trends. For $\tau_{c1}$ , the largest composition-dependent separation occurred at intermediate and high R . At R=0.2 , the 50:50 and biotin–NeutrAvidin networks showed substantially larger $\tau_{c1}$ values than pure α -actinin, with the 50:50 condition approximately three-to four-fold higher than the α -actinin network. At R=0.3 , the 50:50 network had the largest $\tau_{c1}$ , approximately 60–70% higher than pure α -actinin and about 20% higher than biotin–NeutrAvidin. Thus, mixed crosslinking most strongly shifted the onset of the elastic-dominant window at high crosslinker concentration. By contrast, $\tau_{c2}$ was largest for biotin–NeutrAvidin across the measured R values, with the clearest separation at R=0.1 . Thus, the mixed network most strongly affected the first crossover, whereas persistent biotin–NeutrAvidin connectivity most strongly affected the second crossover over the measured range.

Finally, the high-frequency portions of the moduli were fit to power laws, $G^{\prime }\left(\omega \right)∼\omega^{\alpha },\;G^{\prime \prime }\left(\omega \right)∼\omega^{\beta }$ , where α and β describe the frequency dependence of the elastic and viscous responses, respectively (Supplementary Figure S2A,B). These exponents changed more modestly than the crossover times or viscosity. The elastic exponent, α , showed some composition-dependent variation, most clearly at R=0.2 , where the biotin–NeutrAvidin value was approximately 30–40% higher than the pure α -actinin value. In contrast, the viscous exponent, β , remained clustered near the same value across crosslinker identities and R . This indicates that the high-frequency scaling of $G^{\prime \prime }\left(\omega \right)$ was relatively insensitive to crosslinker composition, whereas the elastic scaling of $G^{\prime }\left(\omega \right)$ was more responsive to crosslinker identity.

### 3.3 Crosslinking increases nonlinear resistance to imposed microscale strain

**FIGURE 4:**
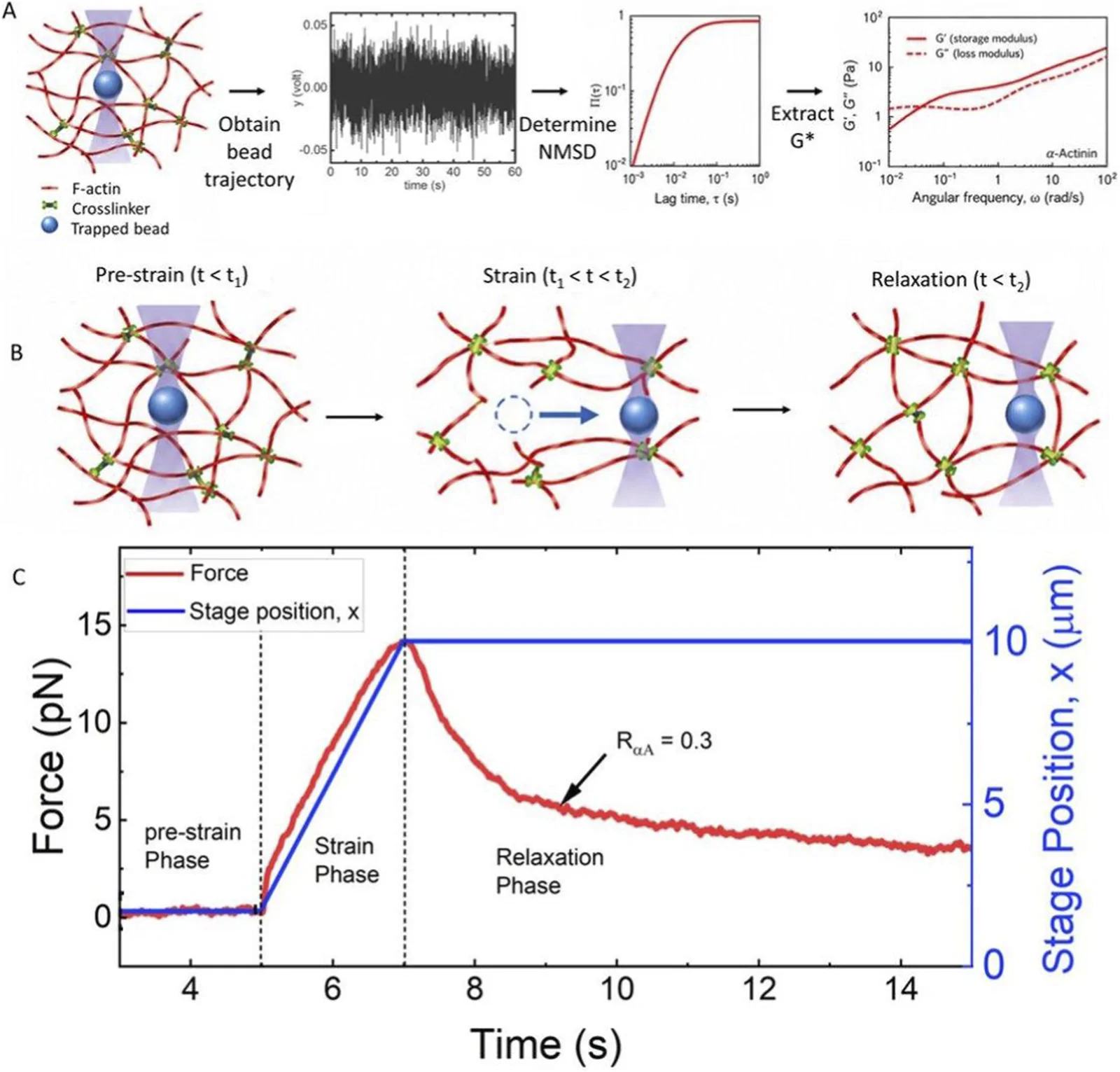
Optical-tweezers microrheology workflow for measuring linear and nonlinear mechanics of crosslinked F-actin networks. **(A)** Passive microrheology analysis. An optically trapped bead embedded in a crosslinked F-actin network undergoes thermal fluctuations that are recorded as a bead-position trajectory, y(t) . The trajectory is converted to the normalized mean-squared displacement, Π(τ) , and generalized Langevin analysis is used to extract the complex shear modulus, $G^{*}\left(\omega \right)=G^{\prime }\left(\omega \right)+iG^{\prime \prime }\left(\omega \right)$ , where $G^{\prime }\left(\omega \right)$ and $G^{\prime \prime }\left(\omega \right)$ report the elastic storage and viscous dissipation of the network, respectively. **(B)** Active microrheology protocol. During the pre-strain phase, $t<t_{1}$ , the bead fluctuates about its equilibrium position. During the strain phase, $t_{1}\leq t\leq t_{2}$ , the stage is translated to displace the bead relative to the surrounding network; the dashed circle marks the initial bead position and the blue arrow indicates the imposed displacement. During relaxation, $t>t_{2}$ , the stage is held fixed while the force decay is measured. **(C)** Representative force and stage-position traces for an α -actinin-crosslinked network at R=0.3 . The blue curve shows the imposed stage displacement, and the red curve shows the resulting network force. The force rises during strain and decays after motion stops, but it does not return to zero within the measurement window, indicating incomplete stress relaxation and residual force after deformation.

We next asked whether the composition-dependent trends observed in the linear regime persisted under large-amplitude deformations that drive the networks out of equilibrium. In these measurements, a trapped microsphere was displaced through the network while the force exerted on the bead was recorded (Figures 4B,C). Because both the probe size (4.2 μ m) and imposed displacement (10 μ m) exceeded the estimated mesh size (0.6 μ m), bead motion required deformation of the surrounding actin network rather than passage through isolated pores. Thus, the nonlinear measurement reports a local network-scale response involving filament recruitment, crosslink loading, and possible yielding or rearrangement of the deformed mesh.

**FIGURE 5:**
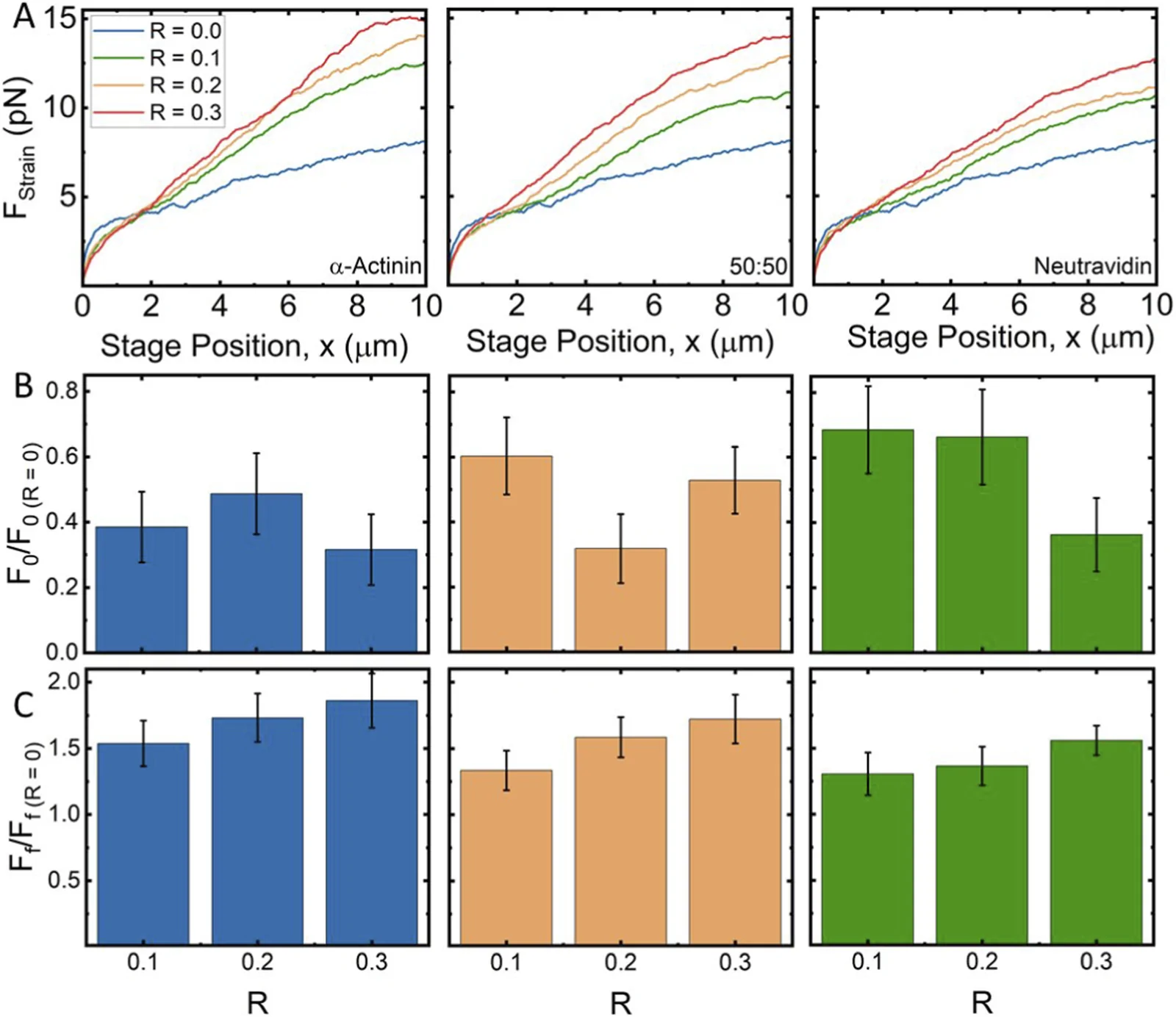
Nonlinear strain force increases with crosslinker concentration for all network compositions. **(A)** Force measured as a function of stage displacement as a trapped microsphere is pulled through F-actin networks crosslinked with pure α -actinin, 50:50 α -actinin/biotin–NeutrAvidin, or pure biotin–NeutrAvidin. For all three crosslinker compositions, the force increases with displacement and with the total crosslinker ratio R , indicating that increased crosslinking increases resistance to imposed microscale deformation. **(B)** Initial strain force, $F_{0}$ , normalized to the corresponding uncrosslinked network value. **(C)** Terminal strain force, $F_{f}$ , normalized to the uncrosslinked control. In panels B and C, blue denotes pure α -actinin networks, orange denotes 50:50 α -actinin/biotin–NeutrAvidin networks, and green denotes pure biotin–NeutrAvidin networks. The initial-force response is less monotonic than the terminal-force response, indicating that early resistance is sensitive to local probe environment and network heterogeneity. The terminal force increases more systematically with R , showing that crosslinking most strongly affects the accumulated resistance during large local deformation. At comparable R , α -actinin networks generally produce the largest terminal forces, biotin–NeutrAvidin networks produce the smallest forces, and 50:50 networks show intermediate responses. Panels B and C show only the crosslinked networks (R>0) . All values are normalized to the corresponding uncrosslinked (R=0) actin-network values; consequently, the R=0 reference has a normalized value of one and is omitted from the plots for clarity.

For all three crosslinker compositions, the strain force increased with stage displacement (Figure 5A). This rising force indicates that the network resisted bead motion increasingly as the probe moved through and deformed the surrounding actin mesh. The force also generally increased with increasing R , showing that added crosslinks increased resistance to imposed microscale deformation. This trend was clearest in the terminal force, $F_{f}$ , at the end of the strain phase, where larger R produced larger normalized final force, $F_{f}/F_{f}\left(R=0\right)$ , for all three crosslinker conditions (Figure 5C).

At comparable R , pure α -actinin networks generally exhibited the largest terminal forces, pure biotin–NeutrAvidin networks exhibited the smallest forces, and 50:50 networks showed intermediate responses. Thus, biotin–NeutrAvidin linkages did not inherently produce the largest nonlinear strain force. The initial-force summaries, $F_{0}/F_{0}\left(R=0\right)$ , were less monotonic than the terminal-force summaries, $F_{f}/F_{f}\left(R=0\right)$ (Figure 5B), indicating that early resistance was more sensitive to the local probe environment, whereas terminal force reflected the accumulated response of a larger deformed network region.

Overall, increasing R increased resistance to large-amplitude strain within each crosslinker composition, while crosslinker identity determined the magnitude of that resistance. The 50:50 condition remained largely intermediate in total strain force, reinforcing the conclusion that mixed crosslinking does not uniformly maximize mechanical response across all observables.

### 3.4 Differential modulus reveals a non-monotonic mixed-crosslinker stiffening response

To determine how network stiffness evolved during nonlinear strain, we calculated the differential modulus, K=dF/dx , from the force–displacement curves in Figure 5A. Whereas total strain force measures accumulated resistance, K reports the instantaneous change in force with bead displacement and is therefore more sensitive to stiffening, softening, and yielding.

**FIGURE 6:**
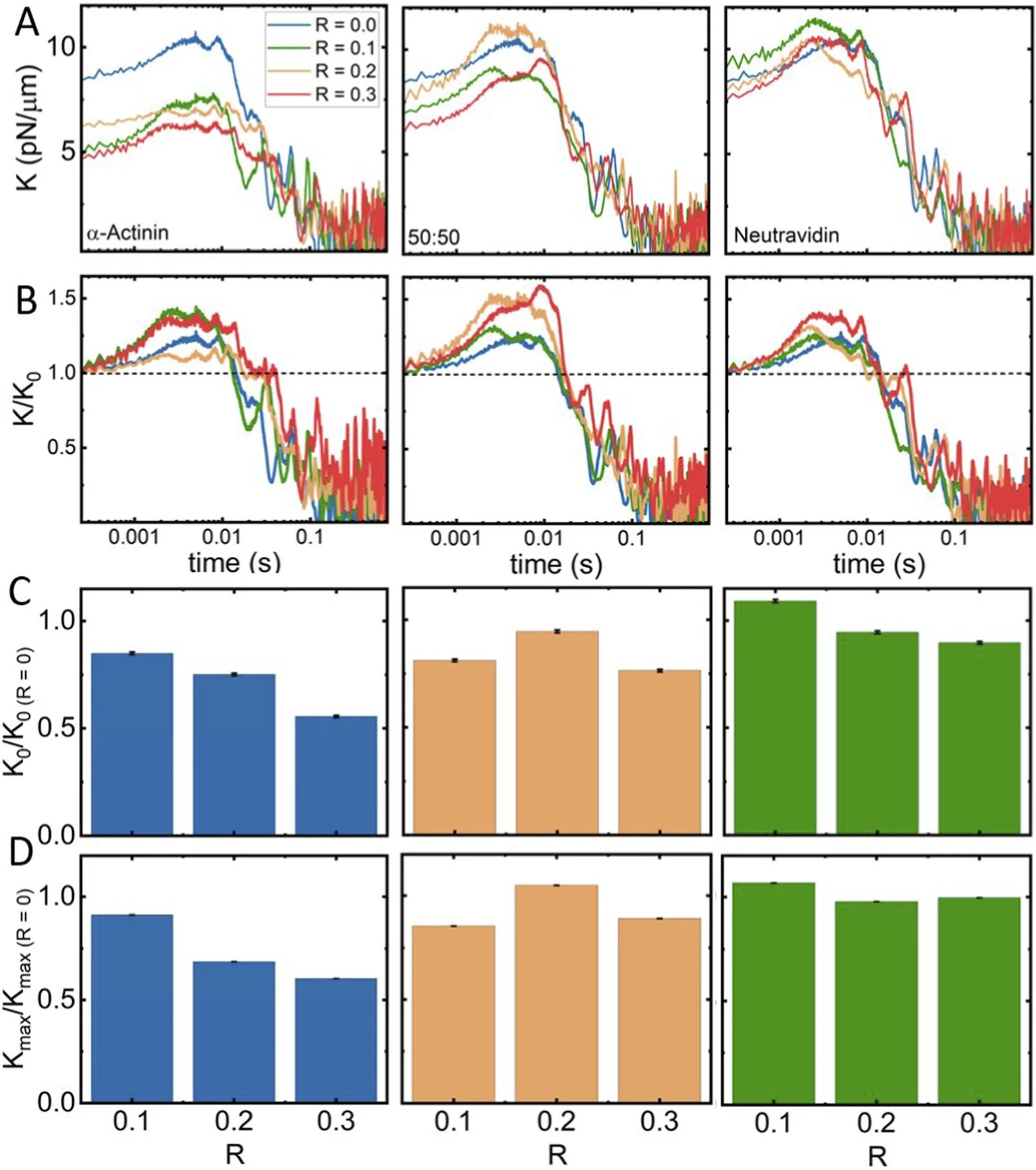
Differential modulus reveals transient stiffening followed by softening and composition-dependent yielding. **(A)** Differential modulus, K=dF/dx , calculated from the strain-force curves for pure α -actinin, 50:50 α -actinin/biotin–NeutrAvidin, and pure biotin–NeutrAvidin networks. All networks exhibit an early peak in K , followed by a decay at later times, indicating transient stiffening followed by softening or yielding. **(B)** Normalized differential modulus, $K/K_{0}$ , where $K_{0}$ is the initial differential modulus for each condition. The horizontal dotted line at $K/K_{0}=1$ guides the eye and marks the transition between strain stiffening $\left(K/K_{0}>1\right)$ and strain softening $\left(K/K_{0}<1\right)$ . The normalized curves rise above unity at early times and then fall below unity as the network yields. **(C)** Initial differential modulus, $K_{0}$ , normalized to the uncrosslinked control. **(D)** Maximum differential modulus, $K_{\max}$ , normalized to the uncrosslinked control. In panels C and D, blue denotes pure α -actinin networks, orange denotes 50:50 α -actinin/biotin–NeutrAvidin networks, and green denotes pure biotin–NeutrAvidin networks. Pure α -actinin networks show decreasing $K_{0}$ and $K_{\max}$ with increasing R , while biotin–NeutrAvidin networks vary more weakly. The 50:50 networks show a distinct non-monotonic response, with both $K_{0}$ and $K_{\max}$ peaking at intermediate R=0.2 , indicating that mixed crosslinking can produce nonlinear mechanical behavior that is not a simple average of the two pure-crosslinker limits. Panels C and D show only the crosslinked networks (R>0) . All values are normalized to the corresponding uncrosslinked (R=0) actin-network values; consequently, the R=0 reference has a normalized value of 1 and is omitted from the plots for clarity.

All networks showed a characteristic nonlinear sequence: an early increase in K , followed by a decrease at later displacements (Figure 6A). In the normalized plots, $K/K_{0}$ rose above unity at short times and then dropped below unity as the bead continued to move (Figure 6B). This behavior indicates transient stiffening followed by softening or yielding to a steady-state regime.

The dependence of $K_{0}$ and $K_{\max}$ on R was strongly composition-dependent (Figure 6C,D). For pure α -actinin networks, both the initial differential modulus and maximum differential modulus decreased as R increased, even though the total strain force increased with R . Pure biotin–NeutrAvidin networks showed weaker variation in both $K_{0}$ and $K_{\max}$ over the range tested. In contrast, the 50:50 networks showed the most distinctive behavior: both $K_{0}$ and $K_{\max}$ peaked at intermediate R=0.2 , rather than increasing or decreasing monotonically.

This non-monotonic differential-modulus response provides the clearest mechanical evidence that the mixed-crosslinker system is not simply a linear average of the two pure-crosslinker networks. At intermediate crosslinker concentration, the combination of dynamic α -actinin and persistent biotin–NeutrAvidin crosslinks produced the strongest transient stiffening under local deformation. At lower R , the network may be insufficiently connected to recruit a large load-bearing region, whereas at higher R , increased heterogeneity or over-constrained connectivity may promote local yielding and reduce the normalized differential stiffening response.

### 3.5 Post-strain relaxation reveals crosslinker-dependent stress retention

After the strain phase, the stage was held fixed and the force exerted by the deformed network was monitored over time. This relaxation phase reports how the network dissipates the stress generated during bead displacement and whether it returns toward its initial force state or retains residual stress.

**FIGURE 7:**
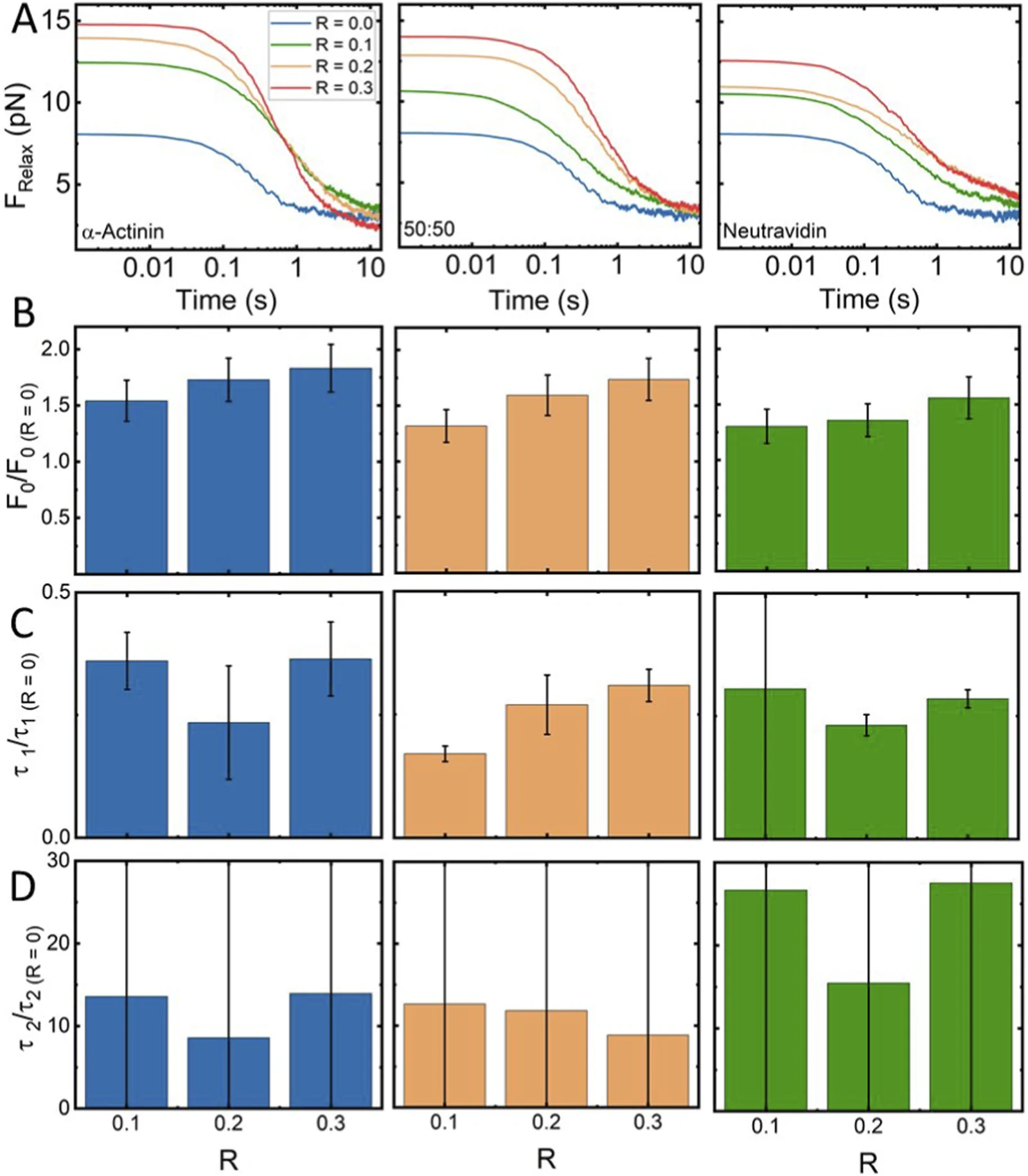
Post-strain force relaxation separates dynamic and effectively permanent crosslinking responses. **(A)** Force relaxation after the imposed strain for pure α -actinin, 50:50 α -actinin/biotin–NeutrAvidin, and pure biotin–NeutrAvidin networks. All networks relax after the stage motion stops, but none return fully to zero force within the measurement window, indicating residual stress and incomplete structural recovery. Pure α -actinin networks show the steepest early decay, consistent with faster stress relaxation mediated by dynamic crosslinker unbinding and rebinding. Biotin–NeutrAvidin networks relax more slowly and retain larger late-time forces, consistent with more persistent crosslinks. The 50:50 networks generally exhibit intermediate relaxation behavior. **(B)** Initial relaxation force normalized to the uncrosslinked control, showing that the force present immediately after strain increases with R . **(C,D)** Characteristic relaxation times obtained from exponential fits to the force-decay curves. Because the long-time fits show large uncertainty, especially for $\tau_{2}$ , the direct force-decay trends in panel A provide the most robust comparison of relaxation behavior across crosslinker compositions. In panels B, C, and D, blue denotes pure α -actinin networks, orange denotes 50:50 α -actinin/biotin–NeutrAvidin networks, and green denotes pure biotin–NeutrAvidin networks. Panels B–D show only the crosslinked networks (R>0) . All values are normalized to the corresponding uncrosslinked (R=0) actin-network values; consequently, the R=0 reference has a normalized value of one and is omitted from the plots for clarity.

All networks relaxed after the strain ended, but none returned fully to zero force within the measurement window (Figure 7A). This incomplete relaxation indicates that bead displacement produced both recoverable elastic stress and irreversible or slowly reversible structural remodeling. The persistence of a nonzero force at late times suggests that the networks retained mechanical memory of the imposed deformation.

Relaxation behavior differed systematically across crosslinker compositions. Pure α -actinin networks showed the steepest force decay, indicating the fastest stress relaxation. Pure biotin–NeutrAvidin networks relaxed more gradually and retained larger late-time forces, consistent with greater stress retention. The 50:50 networks generally displayed intermediate relaxation behavior, retaining partial stress while still providing relaxation pathways.

The initial relaxation force increased with R for all three compositions (Figure 7B), consistent with the larger terminal forces (Figure 5C) measured during the strain phase. Networks with more crosslinks accumulated larger stresses during deformation and therefore began the relaxation phase from a higher force. However, the subsequent decay depended strongly on crosslinker identity.

To compare stress dissipation directly, we determined the fraction of force retained at the end of the relaxation window. We define the retained fraction as $F_{\text{rel},15}/F_{\text{rel},0}$ , where $F_{\text{rel},0}$ is the force at the start of relaxation and $F_{\text{rel},15}$ is the force at t≈15 s . The corresponding percent decay is $100\times \left[1-F_{\text{rel},15}/F_{\text{rel},0}\right]$ . Here, $F_{\text{rel},0}$ and $F_{\text{rel},15}$ were read directly from the relaxation curves shown in Figure 7A for each condition.

For pure α -actinin networks, increasing R increased the initial post-strain force but decreased the fraction of force retained after relaxation. The R=0 control retained roughly 35–40% of its initial relaxation force, corresponding to a decay of about 60–65% . In contrast, α -actinin-crosslinked networks retained progressively less force with increasing R : approximately 25–30% at R=0.1 , 20–25% at R=0.2 , and 15–20% at R=0.3 . Thus, the highest- R α -actinin networks dissipated nearly 80–85% of the initial relaxation force within the measurement window.

The 50:50 mixed networks showed an intermediate but distinct relaxation response. Their initial relaxation force increased with R , as in the pure-crosslinker networks, but their late-time force remained comparatively clustered near ∼3 pN . As a result, the retained fraction decreased from roughly 30% at R=0.1 to about 20–25% at R=0.2 and R=0.3 , corresponding to a force decay of approximately 70–80% . Pure biotin–NeutrAvidin networks retained the largest fraction of their initial force across the crosslinked conditions. For R=0.1–0.3 , the late-time force remained approximately 30–35% of the initial post-strain force, corresponding to a decay of ∼65–70% .

To further characterize relaxation dynamics, we fit the force decay to a two-mode exponential form, $F\left(t\right)=C_{1}e^{-t/\tau_{1}}+C_{2}e^{-t/\tau_{2}}+F_{R},$ where $F_{R}$ is the residual force. Across network conditions, this form captured both fast and slow components of post-strain relaxation (Figure 7C,D). The fitted fast timescale, $\tau_{1}$ , remained below the R=0 value for nearly all crosslinked networks, typically falling in the range of $∼0.2\text{–}0.4\;\tau_{1}\left(R=0\right)$ . In contrast, the fitted slow timescale, $\tau_{2}$ , was larger than the R=0 value for crosslinked networks, reaching approximately $8\text{–}14\;\tau_{2}\left(R=0\right)$ for α -actinin and 50:50 networks and as much as $∼15\text{–}27\;\tau_{2}\left(R=0\right)$ for biotin–NeutrAvidin networks. Because the long-time fits carry substantial uncertainty, especially for $\tau_{2}$ , the direct force-decay trends and residual-force fractions provide the most robust comparison of relaxation behavior across crosslinker compositions.

## 4 Discussion

The combined imaging, linear microrheology, and nonlinear microrheology measurements show that crosslinker identity and lifetime decouple different aspects of F-actin network mechanics. Rather than producing a simple hierarchy in which one crosslinker is always weakest, the other is always strongest, and the mixed network is uniformly enhanced, α -actinin and biotin–NeutrAvidin tune distinct mechanical features. The outcome depends on the observable, the crosslinker concentration, and the deformation regime being probed. A limitation of the present study is that the mixed-crosslinker networks were examined at one equimolar α -actinin/biotin–NeutrAvidin composition. Therefore, the results establish how this representative 50:50 mixture differs from the single-crosslinker limits at the same nominal total R , but they do not define the full dependence of network architecture or mechanics on crosslinker mixing ratio. Future measurements at fixed total R , for example, 25:75, 50:50, and 75:25 α -actinin:NeutrAvidin compositions at R=0.2 , will be needed to determine whether the observed mixed-network behavior reflects cooperative coupling, a non-monotonic composition dependence, or dilution of each individual crosslinking mechanism.

Structurally, the mixed 50:50 networks showed their strongest distinction at high R , where they developed the largest characteristic structural correlation length. This result is consistent with prior work showing that actin crosslinkers can produce structural polymorphism rather than simply increasing network connectivity. α -Actinin-crosslinked networks, for example, can form structures ranging from relatively uniform networks to bundled or clustered states depending on crosslinker concentration and assembly conditions (Wachsstock et al., 1993; Lieleg et al., 2009; Falzone et al., 2012; Dwyer et al., 2022; Nkusi et al., 2025). In the present system, the mixed network appears to occupy a distinct mesoscale state in which dynamic α -actinin-mediated rearrangement and persistent biotin–NeutrAvidin stabilization together promote spatial heterogeneity, bundled domains, and larger voids. Importantly, this structural effect is concentration-dependent rather than monotonic across all R . A second limitation is that the spatial distributions of α -actinin and NeutrAvidin were not measured directly in the mixed networks. The confocal images report the organization of phalloidin-labeled F-actin, but they do not reveal whether the two crosslinking species are uniformly colocalized or partially segregated into distinct local domains. The repeated strain-force responses shown in Supplementary Figures S3, S4 indicate that the 50:50 networks were mechanically reproducible across bead-pull trials and did not exhibit clearly bimodal force-response populations suggestive of large-scale mechanical segregation. Nevertheless, these measurements cannot rule out crosslinker segregation at smaller length scales. We therefore interpret the 50:50 condition as a network prepared with equal nominal contributions of α -actinin and biotin–NeutrAvidin, rather than as a directly verified uniform mixture of the two crosslinkers. Future experiments using dual-color labeled α -actinin and NeutrAvidin would be needed to directly quantify crosslinker colocalization and determine whether local crosslinker segregation contributes to the observed mesoscale heterogeneity.

In the linear regime, this structural coarsening did not translate into uniform enhancement of viscoelasticity. Pure α -actinin networks produced the largest and most concentration-sensitive increases in $G^{\prime }\left(\omega \right)$ , $G^{\prime \prime }\left(\omega \right)$ , and $\eta_{\text{eff}}\left(\omega \right)$ , whereas biotin–NeutrAvidin networks showed more clustered and weakly R -dependent curves. The 50:50 networks generally fell between these limits. These trends place an important boundary on the interpretation of crosslinker cooperation: the mixed network is not simply more solid-like than either pure-crosslinker network in the small-amplitude linear regime. Instead, linear viscoelasticity appears to be governed by how each crosslinking scheme controls both network connectivity and accessible stress-relaxation pathways.

The crossover-time and power-law analyses further support this interpretation. The first crossover time was most strongly shifted by the 50:50 condition at high R , indicating that mixed crosslinking can alter the timescale over which the network enters an elastic-dominant response. In contrast, the second crossover time was largest for biotin–NeutrAvidin, consistent with persistent crosslinks extending the timescale over which stored stress remains constrained. However, these crossover times should be interpreted as apparent mechanical timescales rather than direct measures of a single molecular process. In entangled polymer systems, crossovers between $G^{\prime }\left(\omega \right)$ and $G^{\prime \prime }\left(\omega \right)$ are often associated with transitions among terminal viscous relaxation, an intermediate elastic-dominant regime, and short-time local dynamics (Doi and Edwards, 1988; Falzone et al., 2015; Gurmessa et al., 2016; Pinchiaroli et al., 2024). In crosslinked actin networks, filament entanglements, network architecture, and crosslinker binding lifetimes all contribute to stress storage and relaxation. Thus, shifts in crossover time reflect the combined influence of polymer dynamics and crosslink-mediated connectivity.

The nonlinear measurements revealed a different aspect of crosslinker cooperation. During bead displacement, all crosslinked networks showed increasing force with increasing R , but the mixed networks did not produce the largest total force. Pure α -actinin networks generally produced the largest terminal forces, while pure biotin–NeutrAvidin networks produced the smallest. This indicates that nonlinear force generation is not determined by crosslink lifetime alone. Although biotin–NeutrAvidin linkages are more persistent, persistence does not guarantee a larger force if the resulting network architecture does not recruit filaments efficiently into the load-bearing region around the moving bead. Conversely, α -actinin networks may generate larger accumulated forces if their architecture couples more effectively to the probe during large local deformation.

The clearest emergent mechanical signature of the mixed network appeared in the differential modulus. The 50:50 networks exhibited a non-monotonic peak in both $K_{0}$ and $K_{\max}$ at R=0.2 , whereas the pure-crosslinker networks showed weaker or monotonic trends. This indicates that mixed crosslinking can optimize transient nonlinear stiffening at an intermediate crosslinker density, even when it does not maximize linear moduli or total strain force. Such behavior is consistent with nonlinear mechanics in crosslinked semiflexible polymer networks, where stiffening can arise from filament alignment, tension buildup, and load-bearing network recruitment, while subsequent softening can arise from crosslinker unbinding, filament rearrangement, bundle deformation, or local yielding (Gardel et al., 2006; Tharmann et al., 2007; Gurmessa et al., 2017; Yang et al., 2013; Kim et al., 2014; Plagge et al., 2016). In the mixed networks, dynamic crosslinks may facilitate rearrangement and filament recruitment, while persistent crosslinks stabilize selected load-bearing contacts. At intermediate R , these effects may be optimally balanced; at high R , coarsening or over-constrained connectivity may promote local yielding and reduce differential stiffening.

Post-strain relaxation further distinguished the mechanical roles of the two crosslinking schemes. Pure α -actinin networks relaxed most rapidly and dissipated the largest fraction of their initial relaxation force at high R , consistent with stress release through reversible crosslink exchange and local network rearrangement. Biotin–NeutrAvidin networks retained a larger fraction of force, consistent with long-lived constraints that restrict filament sliding and preserve deformation-induced stress. The 50:50 networks balanced these limits: they retained some residual stress while still providing relaxation pathways. This behavior suggests that dynamic and persistent crosslinks contribute complementary functions, with α -actinin promoting stress dissipation and biotin–NeutrAvidin promoting mechanical memory.

Comparing the linear and nonlinear measurements also clarifies why the effects of crosslinker lifetime are observed in some regimes but not others. Reported α -actinin bond lifetimes are on the order of fractions of a second to several seconds, depending on isoform and conditions (Wachsstock et al., 1994; Xu et al., 1998). The nonlinear strain phase occurs over approximately 2 s , while the relaxation phase is monitored over approximately 15 s . Thus, α -actinin crosslinks may behave as partially persistent during rapid strain but dynamically exchange during relaxation. By contrast, biotin–NeutrAvidin linkages remain effectively persistent over both timescales. In passive microrheology, small thermal fluctuations probe near-equilibrium stress storage and dissipation over a broad frequency range, where reversible α -actinin exchange can contribute to both elasticity and viscous loss. In active nonlinear microrheology, the imposed deformation recruits network architecture, filament connectivity, and crosslink loading into the response. Therefore, molecular lifetime is important, but it is not the only control parameter; its mechanical influence depends on the timescale and amplitude of the measurement.

These results refine the meaning of synergy in this system. Previous dual-crosslinker studies, including α -actinin/fascin and α -actinin/filamin networks, showed that combinations of actin-binding proteins can enhance particular mechanical properties (Tseng et al., 2005; Esue et al., 2009). Our results are consistent with the broader idea that crosslinkers can cooperate, but they show that cooperation need not appear as a universal increase in stiffness, viscosity, or force. In the α -actinin/biotin–NeutrAvidin system, cooperation is selective and deformation-regime dependent: it is most evident in mesoscale structural heterogeneity at high R and in nonlinear differential stiffening at intermediate R , but not in the overall magnitude of the linear moduli or total strain force.

These findings also suggest a physical mechanism by which cells may tune actin-network mechanics using crosslinkers with different lifetimes. Dynamic α -actinin-like crosslinks can permit local rearrangement, stress dissipation, and remodeling, whereas more persistent constraints can support sustained force transmission and mechanical memory. In contractile actomyosin networks, this balance may allow locally generated myosin forces to be transmitted through stress-bearing structures while still permitting turnover, remodeling, and mechanosensitive adaptation. Because biotin–NeutrAvidin is an engineered model for long-lived crosslinking rather than a physiological crosslinker, we interpret it as a model persistent constraint rather than as a direct molecular analog of a cellular actin-binding protein.

The α -actinin concentrations used here were selected to probe a controlled reconstituted regime in which crosslinker identity and lifetime measurably affect actin-network structure and mechanics. Although these nominal R values cannot be mapped directly onto a unique physiological concentration, because cells regulate crosslinking locally and dynamically, they represent a physically relevant low-to-intermediate *in vitro* regime. We further note that this study is limited to R≤0.3 . At higher R , stronger bundling and greater mesoscale heterogeneity would be expected, but potentially also earlier local yielding or increased sample-to-sample variability. Because this regime was not examined directly, these outcomes should be regarded as predictions rather than conclusions.

A complementary way to interpret our measurements is to compare the entanglement length with the average distance between crosslinks. In crosslinked actin networks, nonlinear microrheology measurements have shown that crosslinking begins to dominate entanglement-mediated dynamics when the distance between crosslinks, $l_{c}$ , becomes smaller than the entanglement length, $l_{e}$ (Gurmessa et al., 2017). Using the nominal geometric estimate $l_{c}\approx l_{\text{mon}}/R$ , where $l_{\text{mon}}\approx 7\;nm=0.007\;\mu m$ is the axial spacing per actin monomer and R is the crosslinker-to-actin ratio, the estimated crosslink spacings for R=0.1 , 0.2, and 0.3 are approximately 0.070 μm , 0.035 μm , and 0.023 μm , respectively. These values are all much smaller than the estimated entanglement length of our actin networks, $l_{e}\equiv \xi_{m}^{4/5}l_{p}^{1/5}∼c_{a}^{-2/5}\approx 1.18\;\mu m,$ where $\xi_{m}$ is the mesh size, $l_{p}\approx 17\;\mu m$ is the actin persistence length, and $c_{a}$ is the actin concentration in mg/mL (Isambert and Maggs, 1996; Maggs and Leibler, 1990). The corresponding ratios are $l_{e}/l_{c}\approx 17$ , 34, and 51 for R=0.1 , 0.2, and 0.3, respectively. Thus, although the actin concentration used here is near but below the regime where entanglements alone are expected to produce robust nonlinear response, the crosslinked samples are expected to lie deep in a crosslink-dominated regime. This supports the interpretation that enhanced stiffening, residual stress, and slow relaxation arise primarily from crosslinker-mediated connectivity and mesoscale network architecture rather than from actin entanglements alone.

The imposed deformation rate further places our measurements in the nonlinear microrheology regime. For a bead diameter D=4.2 μm pulled at v=5 μm/s , the characteristic strain rate is $\dot{\gamma }\approx \frac{3\sqrt{2}v}{D}\approx 5.0\;s^{-1}$ , which is about 1.7-fold larger than the reported nonlinear crossover rate, ${\dot{\gamma }}_{c}∼3\;s^{-1}$ . The probe is also large compared with the relevant actin-network length scales: $D/\xi_{m}\approx 6.9$ , $D/d_{t}\approx 13.5$ , and $D/l_{e}\approx 3.6$ . Here, $\xi_{m}\approx 0.3c_{a}^{-1/2}$ , $d_{t}\equiv \xi_{m}^{6/5}l_{p}^{-1/5}∼c_{a}^{-3/5}$ , $l_{e}\equiv \xi_{m}^{4/5}l_{p}^{1/5}∼c_{a}^{-2/5}$ , where $\xi_{m}$ , $d_{t}$ , and $l_{e}$ are the mesh size, tube diameter, and entanglement length, respectively (Gurmessa et al., 2016; Isambert and Maggs, 1996; Maggs and Leibler, 1990; Odijk, 1983). During a 10 μm displacement, the bead traverses roughly $10/\xi_{m}\approx 16$ mesh sizes, $10/d_{t}\approx 32$ tube diameters, and $10/l_{e}\approx 8.5$ entanglement lengths. Thus, the optical-tweezers measurement probes a collective local network response rather than isolated filament motion. Previous coupled microrheology and filament-tracking measurements further showed that strain-induced filament velocities and displacements decay approximately exponentially with distance from the probe path, with decay lengths of order 10– 18 μm , comparable to the actin persistence length (Gurmessa et al., 2017). This suggests that the imposed microscale strain is spatially localized but can propagate over several microns through connected actin structures. These observable-dependent roles are summarized in Table 2, which highlights how each crosslinking scheme emphasizes a different aspect of network structure, linear response, nonlinear deformation, or stress relaxation.

**TABLE 2:** Summary of crosslinker-dependent structural and mechanical responses.

Response	α -actinin	50:50 mixed	biotin–NeutrAvidin
Mesoscale structure	Relatively finer and more uniform than the mixed network	Most pronounced heterogeneity at high R , with coarser features and larger voids	Relatively uniform, with weaker mesoscale coarsening
Linear viscoelasticity	Largest concentration-dependent increases in $G^{\prime }$ , $G^{\prime \prime }$ , and effective viscosity	Generally intermediate between the two pure-crosslinker limits	More clustered and weakly R -dependent response
Crossover behavior	Smaller apparent shifts in the elastic-dominant window	Largest shift in the first crossover time at high R	Largest second crossover time, consistent with delayed relaxation
Nonlinear force buildup	Generally largest terminal strain force	Intermediate total force response	Generally smallest terminal strain force
Differential stiffening	$K_{0}$ and $K_{\max}$ decrease with increasing R	Non-monotonic response, with $K_{0}$ and $K_{\max}$ peaking at intermediate R	Weaker dependence of $K_{0}$ and $K_{\max}$ on R
Post-strain relaxation	Fastest stress relaxation and greatest dissipation at high R	Intermediate relaxation, balancing dissipation and residual stress	Greater force retention and stronger mechanical memory
Overall interpretation	Dynamic, dissipative, and concentration-sensitive crosslinking	Selective cooperation, most evident in mesoscale structure and nonlinear stiffening	Persistent connectivity and stress retention

The 50:50 mixed network does not uniformly maximize all measured properties. Instead, each crosslinking scheme emphasizes a distinct aspect of F-actin network structure, viscoelasticity, nonlinear stiffening, or stress relaxation.

Taken together, these comparisons show that evaluating actin-network mechanics requires linking structure, linear response, nonlinear deformation, and post-strain recovery. Linear microrheology captures near-equilibrium viscoelasticity, but it does not fully predict how a network responds to large local deformation, where filament recruitment, crosslink loading, and network yielding become central. The α -actinin/biotin–NeutrAvidin system therefore provides a useful model for understanding how cytoskeletal networks combine dynamic adaptability with persistent mechanical memory. In cells, where multiple actin-binding proteins act together, such observable-dependent tuning may be more physiologically relevant than maximizing any single mechanical parameter.

## 5 Conclusion

This study shows that dynamic α -actinin crosslinks and long-lived biotin–NeutrAvidin linkages regulate complementary aspects of F-actin network mechanics. The equimolar mixed α -actinin/biotin–NeutrAvidin networks tested here produced the strongest mesoscale structural heterogeneity at high crosslinker concentration, suggesting that reversible filament rearrangement and persistent contact stabilization can cooperate to promote coarser network organization. Because only the 50:50 composition was examined, these data support distinct behavior of this representative mixed network but do not establish the complete dependence on mixing ratio. However, this structural distinction did not translate into uniformly enhanced linear viscoelasticity; instead, α -actinin produced the strongest concentration-dependent increases in elastic storage, viscous dissipation, and effective viscosity, while biotin–NeutrAvidin showed weaker concentration dependence and mixed networks generally fell between the two pure-crosslinker limits. The clearest emergent behavior of the mixed networks appeared in the nonlinear response, where differential stiffness peaked at an intermediate crosslinker concentration even though the mixed networks did not consistently produce the largest total force. Post-strain relaxation further showed that α -actinin promotes faster stress relaxation, biotin–NeutrAvidin promotes greater force retention, and mixed networks balance these behaviors. Overall, these findings demonstrate that mixed crosslinking does not simply make actin networks uniformly stronger or more solid-like; rather, it decouples structural organization, linear viscoelasticity, nonlinear stiffening, and stress relaxation, providing a mechanism by which cytoskeletal networks can remain mechanically connected while still permitting remodeling under localized forces.

## Data Availability

The original contributions presented in the study are included in the article/Supplementary Material, further inquiries can be directed to the corresponding author.
