# Supplementary material for: Crosslinker identity and dynamics regulate F-actin network structure and mechanics
Source: Front Cell Dev Biol. 2026 Aug 28;14:1900481. doi: 10.3389/fcell.2026.1900481 (PMC13562302; doi:10.3389/fcell.2026.1900481)
Supplement: Supplementary file 1 [file DataSheet1.pdf]

Crosslinker Identity and Lifetime Tune F-Actin Network Structure and Mechanics

Lauren Godfrey and Bekele J. Gurmessa*

Department of Physics and Astronomy, Bucknell University, Lewisburg, PA, USA

Correspondence*:
Bekele Gurmessa
b.gurmessa@bucknell.edu

SUPPLEMENTARY INFORMATION

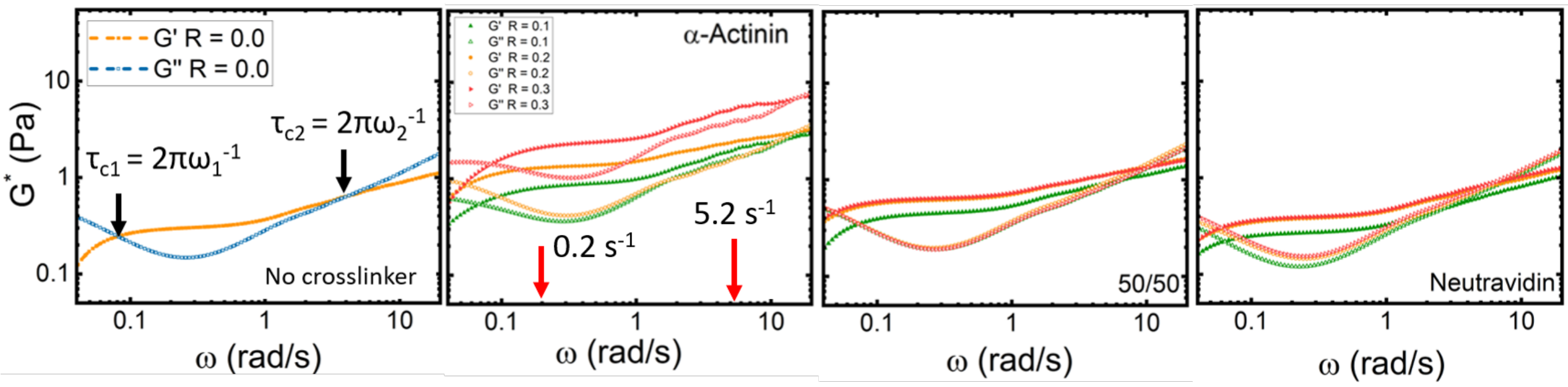

Figure 1. Supplementary Fig. S1. Frequency-dependent linear viscoelastic response of crosslinked F-actin networks. Storage modulus, $G'(\omega)$, shown with closed symbols, and loss modulus, $G''(\omega)$, shown with open symbols, for F-actin networks crosslinked with α-actinin, biotin–NeutrAvidin, or a 50:50 mixture of the two crosslinking schemes. Curves are shown for the indicated total crosslinker-to-actin ratios, R. Vertical arrows at $0.2\ \mathrm{s}^{-1}$ and $5.2\ \mathrm{s}^{-1}$ mark the approximate bounds of the reported α-actinin unbinding-rate window. These markers serve as visual guides to the frequency range over which reversible crosslinker exchange may influence the measured linear viscoelastic response and should not be interpreted as precise crossover frequencies. These data support the crossover-time analysis and high-frequency power-law fits summarized in the main text and Supplementary Fig. S2.

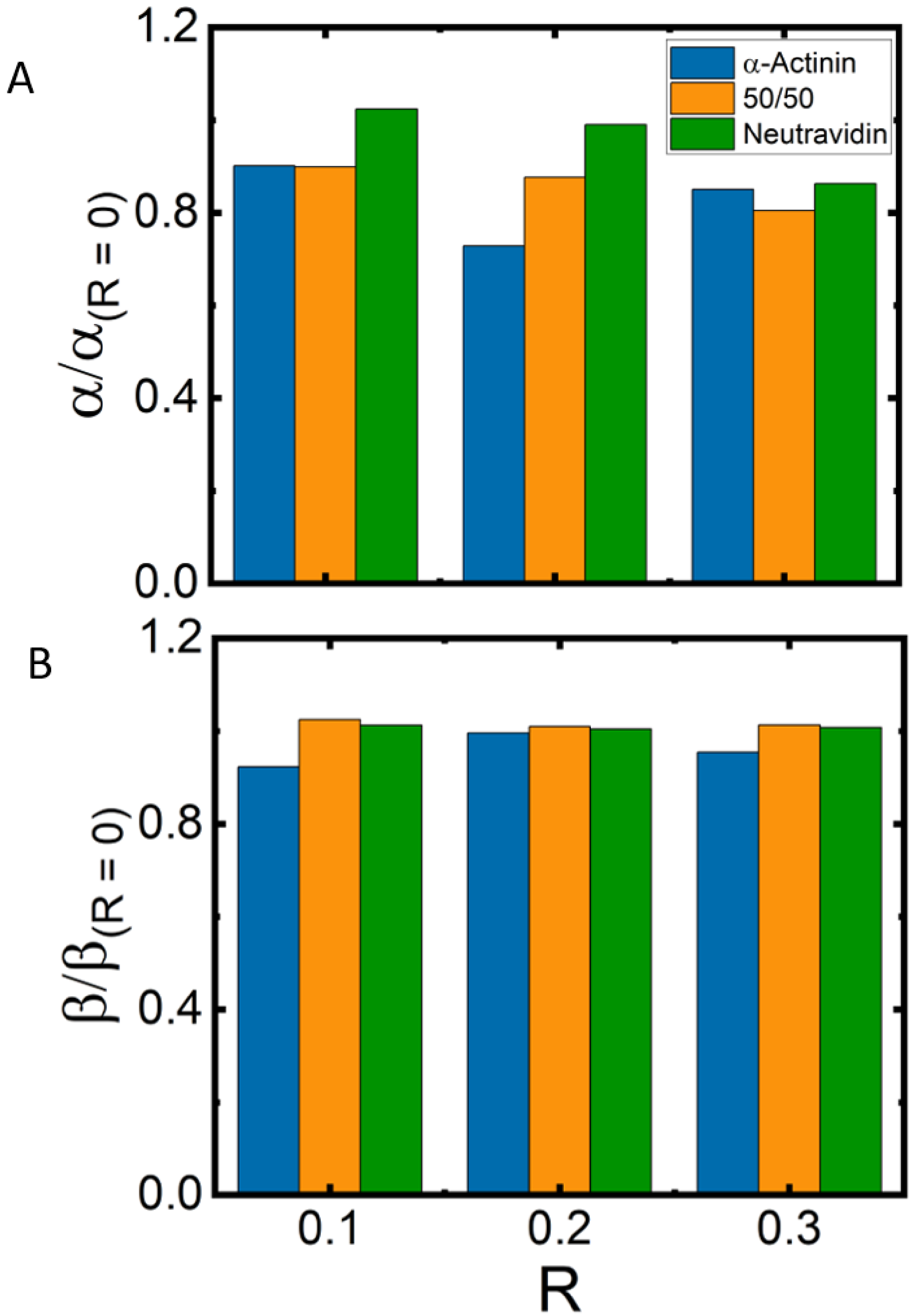

Figure 2. Supplementary Fig. S2. High-frequency power-law scaling of the linear viscoelastic moduli. (A,B) Power-law exponents obtained from high-frequency fits to $G'(\omega) \sim \omega^{\alpha}$ and $G''(\omega) \sim \omega^{\beta}$, respectively, for networks crosslinked with α-actinin, biotin–NeutrAvidin, or a 50:50 mixture. The elastic exponent, α, shows greater sensitivity to crosslinker composition than the viscous exponent, β, indicating that crosslinker identity more strongly affects the high-frequency elastic response than the corresponding viscous scaling.

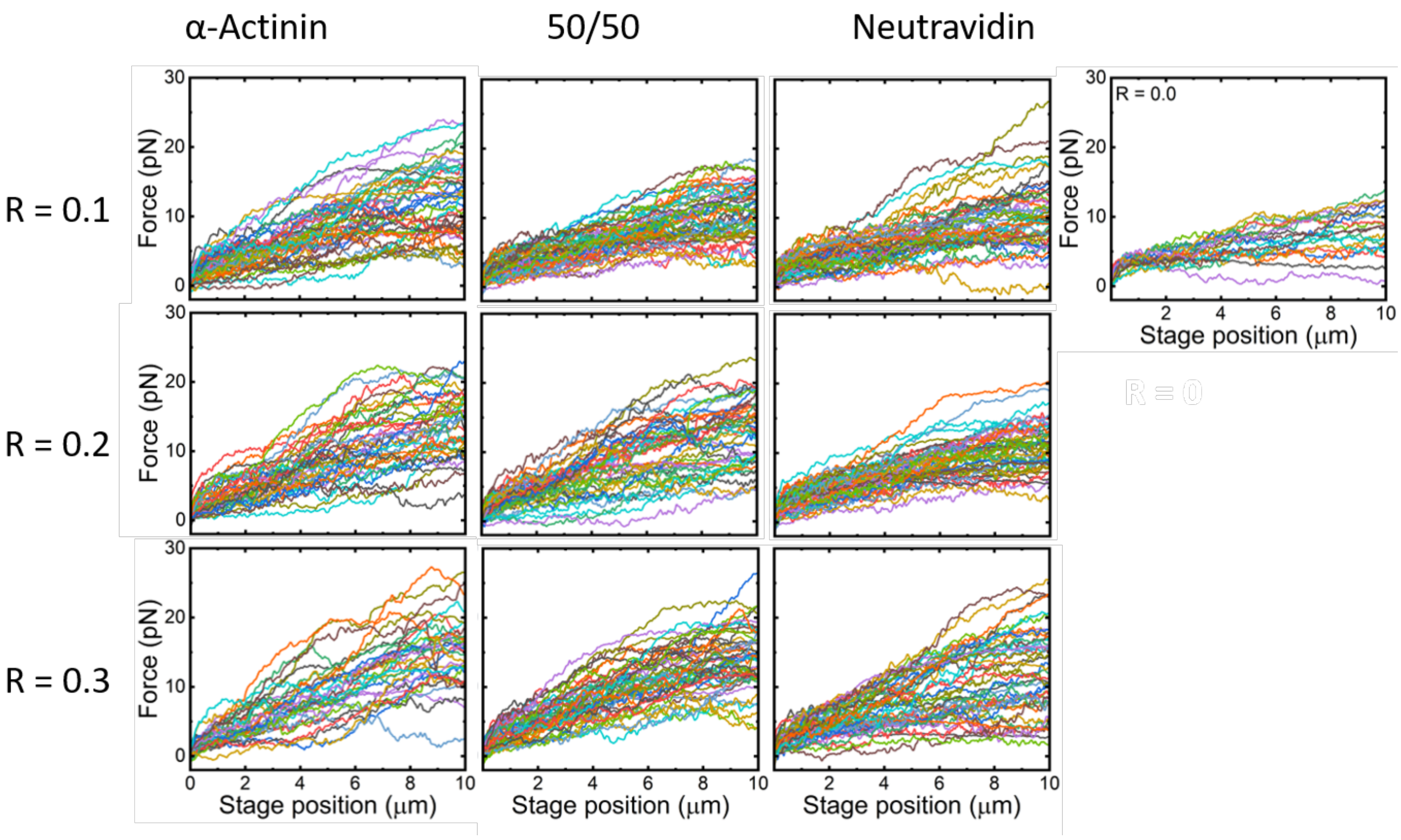

Figure 3. Supplementary Fig. S3. Individual nonlinear force traces during the strain phase. Individual force traces measured while a trapped microsphere was displaced $10\ \mu\mathrm{m}$ through the F-actin networks at $5\ \mu\mathrm{m/s}$. These traces were used to compute the average force–displacement curves shown in Fig. 5A of the main manuscript. Each trial was performed using a different microsphere probe in a different region of the sample chamber. Columns correspond to crosslinker composition, and rows correspond to total crosslinker-to-actin ratio, R, as indicated in the panel labels. The spread among traces reflects local structural heterogeneity and bead-to-bead variation within each network condition.

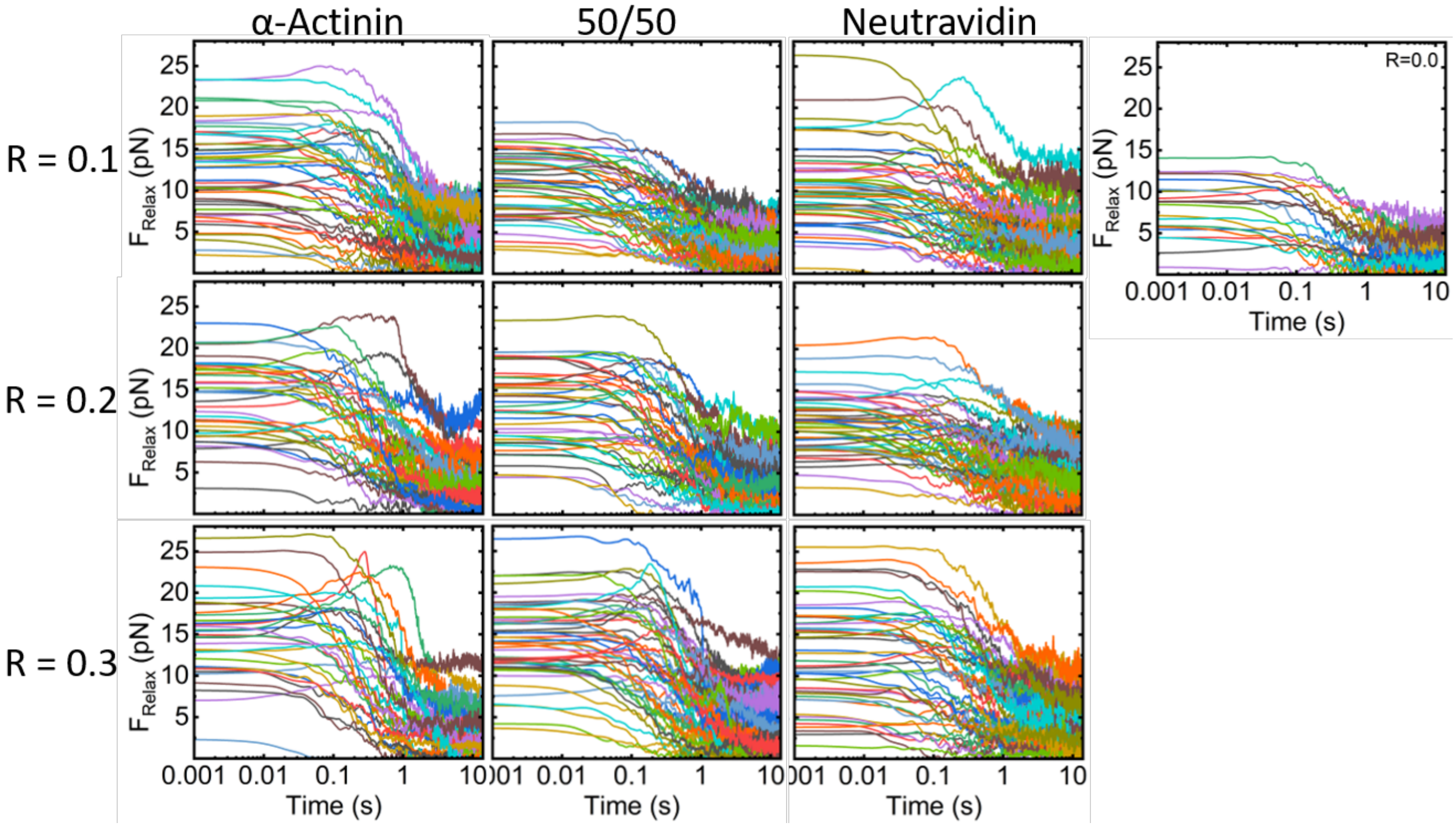

Figure 4. Supplementary Fig. S4. Individual nonlinear force traces during the post-strain relaxation phase. Individual force-relaxation traces measured after the imposed bead displacement ended and the stage was held fixed. These traces were used to compute the average relaxation curves shown in Fig. 7A of the main manuscript. Each trial was performed using a different microsphere probe in a different region of the sample chamber. Columns correspond to crosslinker composition, and rows correspond to total crosslinker-to-actin ratio, R, as indicated in the panel labels. The traces show the trial-to-trial variability underlying the average relaxation response and residual-force analysis reported in the main manuscript.

Table 1. Table S1. Comparison of nominal and mechanistic crosslinking features for α-actinin and biotin–NeutrAvidin actin networks. The nominal crosslinker-to-actin ratio, R, is used as a controlled sample-preparation variable but should not be interpreted as a direct measure of mechanically active crosslink density or effective network connectivity. The molecular architecture, binding dynamics, and actin-crosslinking properties of α-actinin and biotin–NeutrAvidin are summarized based on prior experimental studies (Djinović-Carugo et al., 1999; Wachsstock et al., 1994; Courson and Rock, 2010; Ricketts et al., 2019; Francis et al., 2019).

**Feature**	**α-actinin**	**Biotin–NeutrAvidin**	**50:50 mixed condition**
Nominal R definition	$R_\alpha = [\alpha\text{-actinin}]/[\text{actin}]$	$R_{\text{NA}} = [\text{NeutrAvidin}]/[\text{actin}]$	$R = R_\alpha + R_{\text{NA}}$, with $R_\alpha = R_{\text{NA}} = R/2$
Binding mode	Direct actin-binding protein	Binds biotinylated actin through the biotin–NeutrAvidin interaction	Both binding modes are present
Nominal molecular valency	Typically dimeric actin crosslinker	NeutrAvidin is multivalent for biotin binding, but effective valency depends on biotin-actin availability and steric constraints	Mixed effective valency is not necessarily additive
Binding lifetime	Dynamic and reversible; reported unbinding rates are on the order of $0.2–5.2\ \text{s}^{-1}$	Long-lived and effectively persistent on the experimental timescale	Combines dynamic and persistent linkages
Requirement for modified actin	Does not require biotinylated actin	Requires biotinylated actin	Requires biotinylated actin for the NeutrAvidin component
Potential architectural consequence	Can promote actin bundling and network remodeling in a concentration- and assembly-dependent manner	Forms long-lived crosslinks between biotinylated actin filaments	Combines dynamic and effectively persistent crosslinking; the resulting architecture is determined experimentally in this study
Interpretation of equal R	Equal nominal α-actinin loading	Equal nominal NeutrAvidin loading	Equal total nominal crosslinker loading

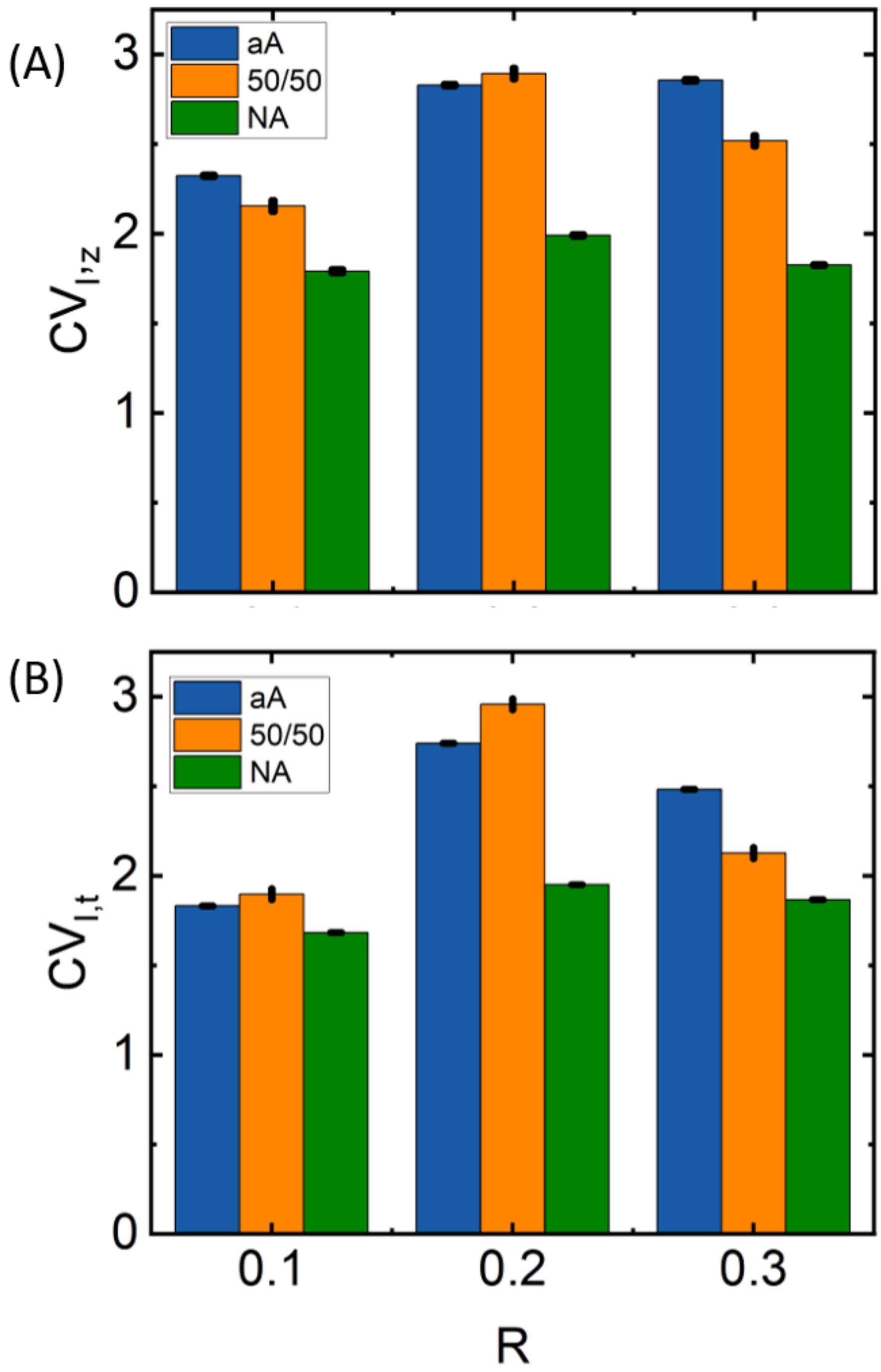

Figure 5. Supplementary Fig. S5. Intensity coefficient of variation analysis of actin-network heterogeneity. Intensity coefficient of variation, $\mathrm{CV}_I = \sigma_I/\langle I\rangle$, was calculated from background-subtracted confocal images as an additional image-based metric of structural heterogeneity. (A) $\mathrm{CV}_{I,z}$ from z-series images and (B) $\mathrm{CV}_{I,t}$ from time-series images for actin networks crosslinked with α-actinin, biotin–NeutrAvidin, or a 50:50 mixture at matched total crosslinker ratios $R = 0.1$, 0.2, and 0.3. Larger CV_I values indicate a broader distribution of fluorescence intensities within the analyzed image. Because CV_I contains no spatial information, it does not directly measure bundle size, pore size, feature spacing, or the spatial arrangement of filament-rich and filament-poor regions. These spatial properties are assessed separately using the autocorrelation lengths ξ_z and ξ_t. Error bars indicate SEM across z-planes or time frames within each stack or video and therefore quantify within-stack or within-video variability rather than independent sample-to-sample variation.

Table 2. Table S2. Summary of structural heterogeneity and nonlinear mechanical parameters used to compare structure–mechanics coupling. Normalized nonlinear mechanical parameters and fluorescence-intensity coefficient of variation metrics are shown for actin networks crosslinked with α-actinin, biotin–NeutrAvidin, or a 50:50 mixture at matched total crosslinker ratio R. Values are reported as mean $\pm$ SEM.

R	Network	$K_0/K_0(R=0)$	$K_{\max}/K_{\max}(R=0)$	$\mathrm{CV}_{I,z}$	$\mathrm{CV}_{I,t}$
0.1	α-actinin	0.8496 ± 0.0062	$0.9132 \pm 3.20 \times 10^{-4}$	2.3229 ± 0.0049	1.8329 ± 0.0008
0.1	50:50	0.8111 ± 0.0061	$0.8550 \pm 2.97 \times 10^{-4}$	2.1535 ± 0.0053	1.8970 ± 0.0011
0.1	biotin–NeutrAvidin	1.0916 ± 0.0075	$1.0880 \pm 3.48 \times 10^{-4}$	1.7904 ± 0.0099	1.6832 ± 0.0007
0.2	α-actinin	0.7511 ± 0.0059	$0.6856 \pm 2.21 \times 10^{-4}$	2.8284 ± 0.0062	2.7402 ± 0.0013
0.2	50:50	0.9459 ± 0.0068	$1.0504 \pm 3.50 \times 10^{-4}$	2.8936 ± 0.0042	2.9590 ± 0.0011
0.2	biotin–NeutrAvidin	0.9459 ± 0.0069	$0.9779 \pm 3.15 \times 10^{-4}$	1.9908 ± 0.0054	1.9508 ± 0.0004
0.3	α-actinin	0.5551 ± 0.0053	$0.6039 \pm 2.09 \times 10^{-4}$	2.8558 ± 0.0066	2.4826 ± 0.0006
0.3	50:50	0.7638 ± 0.0059	$0.8915 \pm 2.89 \times 10^{-4}$	2.5170 ± 0.0042	2.1266 ± 0.0018
0.3	biotin–NeutrAvidin	0.8988 ± 0.0066	$0.9988 \pm 3.53 \times 10^{-4}$	1.8255 ± 0.0037	1.8672 ± 0.0004

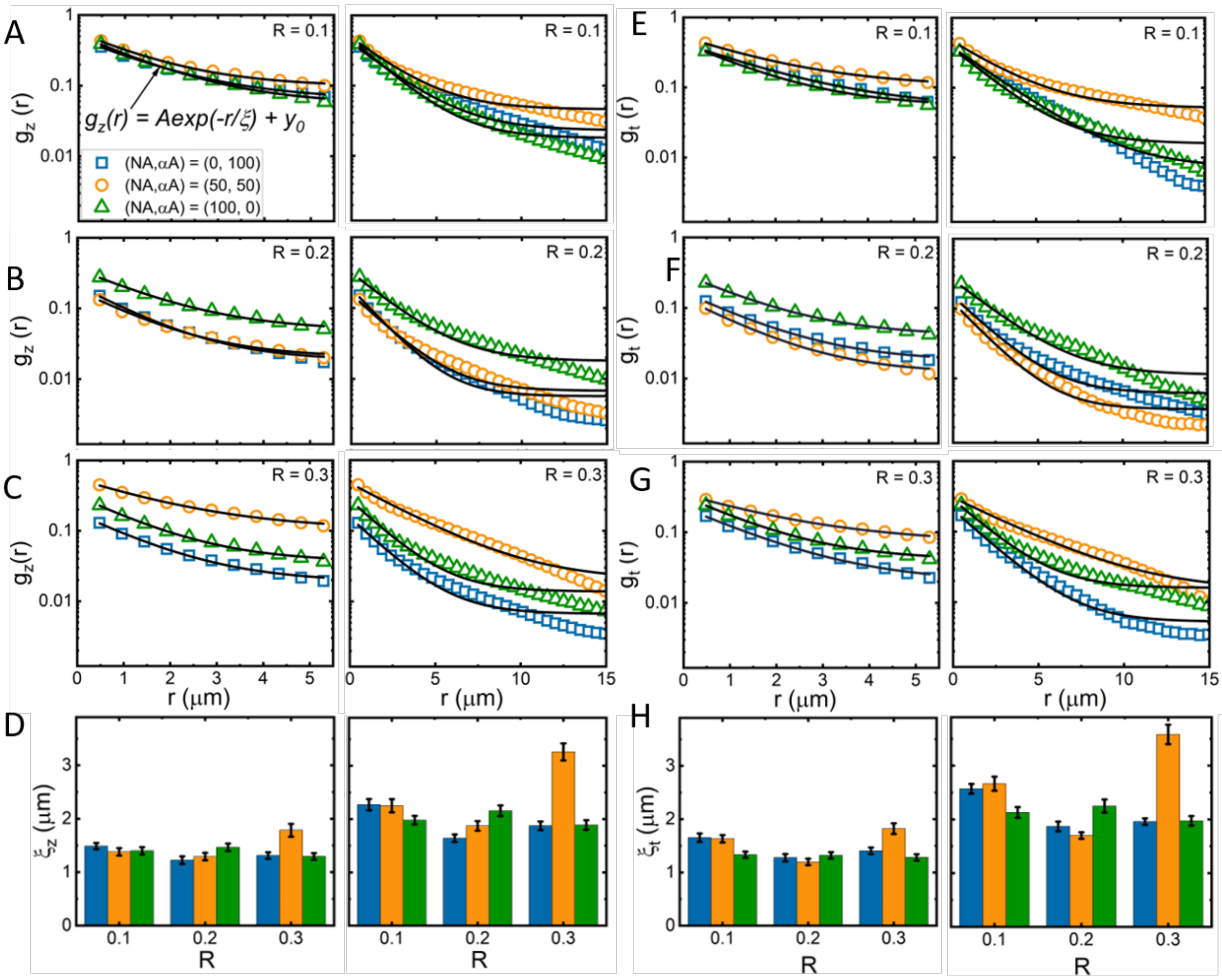

Figure 6. Supplementary Fig. S6. Spatial autocorrelation analysis of crosslinked F-actin networks. Spatial autocorrelation functions obtained from confocal z-stacks (left) and time-stack projections (right) for α-actinin-only, biotin–NeutrAvidin-only, and 50:50 mixed networks at $R = 0.1, 0.2$, and 0.3. For each analysis, the left column highlights the short-distance fitting region ($r < 5$ μm), whereas the adjacent column shows the correlation decay over the extended range $r \leq 15$ μm. Solid curves denote exponential fits of the form $g(r) = A\exp(-r/\xi)$, as illustrated in panel A, used to extract the characteristic correlation lengths ξ_z and ξ_t, summarized in panels D and H, respectively. The $r = 0$ point was excluded from the fits because it corresponds to the trivial self-correlation value, $g(0) = 1$, and is often followed by a sharp initial decrease that can be dominated by pixel-scale effects, finite image resolution, or other sub-resolution features. In the main manuscript, the fits were therefore performed from the first nonzero separation and restricted to the short-distance regime, $r \leq 5$ μm, because the long-distance tails were not consistently well described by a single-exponential decay. The corresponding fit parameters are summarized in Table S3. At $R = 0.3$, the 50:50 mixed network exhibits the largest correlation length in both analyses, consistent with enhanced mesoscale structural heterogeneity.

Table 3. Table S3. Exponential-fit parameters for spatial autocorrelation functions of crosslinked F-actin networks. Characteristic correlation lengths extracted from exponential fits to the z-stack, $g_z(r)$, and time-series, $g_t(r)$, autocorrelation functions over short-range (0.481–5 μm) and extended-range (0.481–15 μm) fitting windows. Values are reported as fit value ± fit uncertainty.

Fit range	R	Condition	ξ_z (μm)	R_z^2	ξ_t (μm)	R_t^2
0.481–5 μm	0.1	α-actinin	1.488 ± 0.060	0.9979	1.653 ± 0.080	0.9983
	0.1	50:50	1.380 ± 0.070	0.9971	1.631 ± 0.070	0.9985
	0.1	biotin–NeutrAvidin	1.398 ± 0.070	0.9974	1.332 ± 0.060	0.9978
	0.2	α-actinin	1.225 ± 0.070	0.9953	1.277 ± 0.070	0.9973
	0.2	50:50	1.292 ± 0.070	0.9952	1.197 ± 0.060	0.9975
	0.2	biotin–NeutrAvidin	1.466 ± 0.070	0.9969	1.321 ± 0.060	0.9982
	0.3	α-actinin	1.311 ± 0.060	0.9973	1.407 ± 0.060	0.9980
	0.3	50:50	1.785 ± 0.120	0.9962	1.823 ± 0.100	0.9980
	0.3	biotin–NeutrAvidin	1.294 ± 0.060	0.9974	1.282 ± 0.060	0.9980
0.481–15 μm	0.1	α-actinin	2.271 ± 0.106	0.9877	2.568 ± 0.090	0.9931
	0.1	50:50	2.251 ± 0.124	0.9826	2.664 ± 0.130	0.9882
	0.1	biotin–NeutrAvidin	1.980 ± 0.080	0.9899	2.129 ± 0.100	0.9861
	0.2	α-actinin	1.642 ± 0.070	0.9872	1.869 ± 0.090	0.9887
	0.2	50:50	1.873 ± 0.090	0.9843	1.699 ± 0.060	0.9900
	0.2	biotin–NeutrAvidin	2.156 ± 0.100	0.9868	2.247 ± 0.122	0.9831
	0.3	α-actinin	1.875 ± 0.080	0.9877	1.960 ± 0.060	0.9934
	0.3	50:50	3.260 ± 0.160	0.9891	3.579 ± 0.180	0.9891
	0.3	biotin–NeutrAvidin	1.889 ± 0.090	0.9863	1.973 ± 0.090	0.9867

REFERENCES

Djinović-Carugo K, Young P, Gautel M, Saraste M. Structure of the alpha-actinin rod: Molecular basis for cross-linking of actin filaments. *Cell* **98** (1999) 537–546.

Wachsstock DH, Schwarz WH, Pollard TD. Cross-linker dynamics determine the mechanical properties of actin gels. *Biophysical Journal* **66** (1994) 801–809.

Courson DS, Rock RS. Actin cross-link assembly and disassembly mechanics for alpha-actinin and fascin. *Journal of Biological Chemistry* **285** (2010) 26350–26357.

Ricketts SN, Francis ML, Farhadi L, Rust MJ, Das M, Ross JL, et al. Varying crosslinking motifs drive the mesoscale mechanics of actin-microtubule composites. *Scientific Reports* **9** (2019) 12831.

Francis ML, Ricketts SN, Farhadi L, Rust MJ, Das M, Ross JL, et al. Non-monotonic dependence of stiffness on actin crosslinking in cytoskeleton composites. *Soft Matter* **15** (2019) 9056–9065.
